# Regioselective Glycosylation of Demethylbellidifolin by Glycosyltransferase AbCGT Yields Potent Anti-Renal Fibrosis Compound

**DOI:** 10.3390/molecules31020309

**Published:** 2026-01-15

**Authors:** Limin Zeng, Shichao Cui, Xingyu Ji, Yuhong Liu, Guozhang Long, Yulan Xia, Gang Cheng, Jingya Li, Youhong Hu

**Affiliations:** 1 School of Chinese Materia Medica, College of Pharmacy, Nanjing University of Chinese Medicine, Nanjing 210023, China; 2 State Key Laboratory of Drug Research, Shanghai Institute of Materia Medica, Chinese Academy of Sciences, Shanghai 201203, China; 3 University of Chinese Academy of Sciences, Beijing 100049, China; 4 School of Pharmaceutical Sciences, Zhejiang Chinese Medical University, Hangzhou 310053, China; 5 Key Laboratory of Glyco-Drug Research of Zhejiang Province, School of Pharmaceutical Science and Technology, Hangzhou Institute for Advanced Study, University of Chinese Academy of Sciences, Hangzhou 310024, China

**Keywords:** glycosyltransferase, regioselectivity, anti-renal fibrosis, biosynthesis, molecular docking

## Abstract

Glycosylation serves as an effective strategy to enhance the solubility, bioavailability, and pharmacological activity of polyhydroxyphenols. In this study, we explored the glycosylation of natural and natural-inspired phenolic compounds using the glycosyltransferase AbCGT and evaluated the anti-renal fibrotic potential of the resulting glycosides. Among them, 1,3,5,8-tetrahydroxyxanthone 5-*O*-β-D-glucopyranoside (**2-1a**), synthesized via the regioselective 5-*O*-glycosylation of demethylbellidifolin, demonstrated significant anti-renal fibrotic activity. In contrast, its homologous glycosyltransferase, UGT73AE1, predominantly glycosylated demethylbellidifolin at the 3-OH position. Molecular docking studies revealed the structural basis for this regioselectivity difference. To enhance the production of **2-1a**, we established a UDP-glucose (UDPG) recycling system by coupling AbCGT with *Glycine max* sucrose synthase (GmSuSy) and subsequently optimized the reaction conditions. Furthermore, targeted mutagenesis of AbCGT informed by molecular docking analysis identified a F138A mutant that enhanced glycosylation yield by 2.3-fold. This work develops a novel glycosyltransferase-based catalytic system and identifies a new compound with potential anti-renal fibrotic activity.

## 1. Introduction

Chronic kidney disease (CKD) has emerged as a global public health challenge [1]. A central pathological feature of its progression is renal fibrosis, particularly tubulointerstitial fibrosis, which ultimately leads to irreversible loss of renal function [2,3]. The transforming growth factor-β (TGF-β) signaling pathway is a central mediator of this fibrogenic process. It acts in concert with other profibrotic factors, such as platelet-derived growth factor (PDGF) and epidermal growth factor (EGF), to activate resident fibroblasts and stimulate the aberrant accumulation of extracellular matrix (ECM) [4]. Although certain advances have been made in understanding the molecular mechanisms, only two antifibrotic drugs, nintedanib and pirfenidone, are currently available for clinical use, both of which exhibit limited efficacy [5,6,7]. To date, no targeted therapy specifically against renal fibrosis has been approved [8,9,10,11], highlighting the urgent need for more effective antifibrotic strategies and agents for CKD.

Glycosylation modification is a crucial biotransformation process in drug development from natural products [12]. The introduction of glycosyl groups at specific sites can significantly enhance drug-like properties, such as aqueous solubility and pharmacokinetic parameters [13,14], and may also modify their bioactivity [15,16,17]. A number of natural glycosides [18,19], including astragaloside IV, ginsenosides, and mangiferin [20,21], exhibit broad anti-fibrotic effects. These compounds act through precise modulation of fibroblast and renal tubular epithelial cell functions, leading to direct inhibition of fibrotic progression [22,23]. These natural products not only serve as valuable lead compounds for drug discovery but also provide critical insights into structure-activity relationships. The clinical success of drugs like dapagliflozin [24], a derivative of the natural flavonoid phlorizin for treating CKD [25,26], further demonstrates the potential of glycoside-based therapeutics in renal medicine.

Glycosyltransferases (GTs) are enzymes that catalyze the transfer of glycosyl groups from activated sugar donors (e.g., UDPG) to acceptor molecules [27]. GTs exhibit high regio- and stereoselectivity in glycosidic bond formation [28]. Conventional chemical glycosylation often involves long and complex synthetic steps, including protection and deprotection steps [29]. Enzymatic catalysis exhibits distinct advantages [30], including environmental friendliness, high efficiency, and unique specificity. However, plant-derived GTs frequently face practical limitations, such as low catalytic activity, narrow substrate scope, and challenges in heterologous expression. Directed evolution or structure-guided rational design of GTs could further optimize their catalytic performance [31,32], enabling tailored glycosylation of diverse pharmacophores.

The glycosyltransferase AbCGT [33], identified from *Aloe barbadensis*, has shown the activity to catalyze *C*-, *O*-, *S*-, and *N*-glycosylation of phenolic and flavonoid substrates. We investigated AbCGT’s glycosylation of polyhydroxyphenolic compounds and identified 1,3,5,8-tetrahydroxyxanthone 5-*O*-β-D-glucopyranoside (**2-1a**) with significant anti-renal fibrotic activity. Molecular docking studies revealed the structural basis for AbCGT’s distinct 5-OH regioselectivity relative to the homologous enzyme UGT73AE1 [34], identifying key active-site interactions that govern site preference. Production of **2-1a** was further enhanced by reaction optimization, a UDPG recycling system with soybean sucrose synthase (GmSuSy) [35], and an engineered AbCGT mutant with better efficiency.

## 2. Results and Discussion

### 2.1. Substrate Screening of AbCGT Against Polyhydroxyphenolic Compounds and Anti-Renal Fibrosis Activity Evaluation of the Glycosylated Products

**Figure 1:**
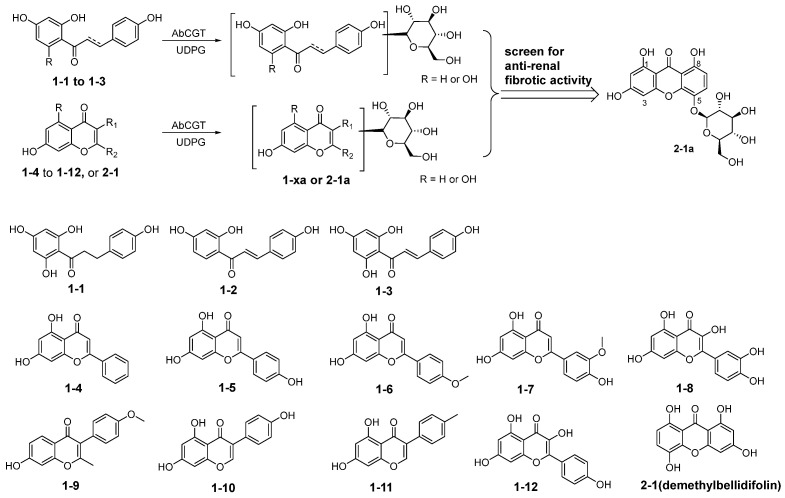
Activity-guided discovery of a potent anti-renal fibrotic glycoside through AbCGT-catalyzed glycosylation followed by functional screening of products.

To investigate the substrate spectrum of AbCGT, we selected 13 structurally diverse polyhydroxyphenolic compounds to produce the corresponding glucosides. The substrates from **1-1** to **1-12** and **2-1** were listed in Figure 1. Using UDPG as the glycosyl donor, the catalytic reactions were performed with purified recombinant AbCGT. The resulting main products, from **1-1a** to **1-12a** and **2-1a,** were subsequently separated by semi-preparative HPLC. Compound **1-1a** was characterized as phloretin-di-*C*-glucoside by NMR. Compounds **1-3a** and **1-6a** were verified as *C*-glycosides by MS/MS analysis through the characteristic loss of 90 and 120 amus (Supplementary Information Figure S2). While the other glucosides were identified as *O*-glucosides due to the loss of one glucose moiety (loss of 162 amus) in MS/MS analysis (Figures S18–S29).

**Figure 2:**
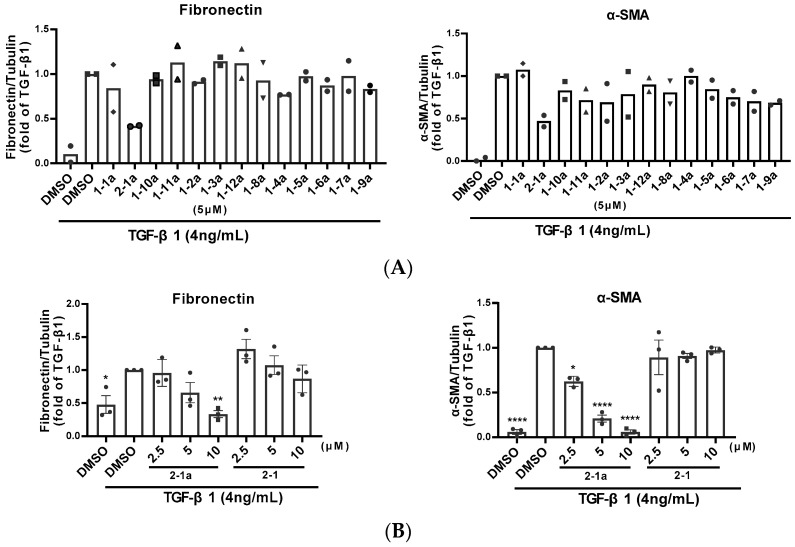
(**A**) The glycosides inhibit protein expression of fibronectin and α-smooth muscle actin (α-SMA). NRK49F cells are preincubated with the glycosides (5 μM) for 1 h before TGF-β1 (4 ng/mL) treatment. Cells are harvested 24 h after TGF-β1 stimulation. Whole cell lysates were prepared and subjected to Western blot analyses. (**B**) Inhibitory effects of glycoside **2-1a**, compared with its aglycone **2-1**. Datas are displayed as means ± SEMs. * *p* < 0.05, ** *p* < 0.01, **** *p* < 0.0001.

All glycosides as the single compound were subsequently evaluated for their anti-renal fibrotic activity as shown in Figure 2A. Notably, the glycosylated product **2-1a,** derived from demethylbellidifolin (**2-1**), demonstrated the most potent anti-renal fibrosis activity by suppressing TGF-β1-induced fibronectin and α-SMA expression. Compared to its aglycone counterpart **2-1**, glycoside **2-1a** showed significantly enhanced inhibitory effects with a dose-dependent response, indicating that glycosylation modification is crucial for its improved bioactivity (Figure 2B).

### 2.2. Structural Confirmation of Product ***2-1a***

**Figure 3:**
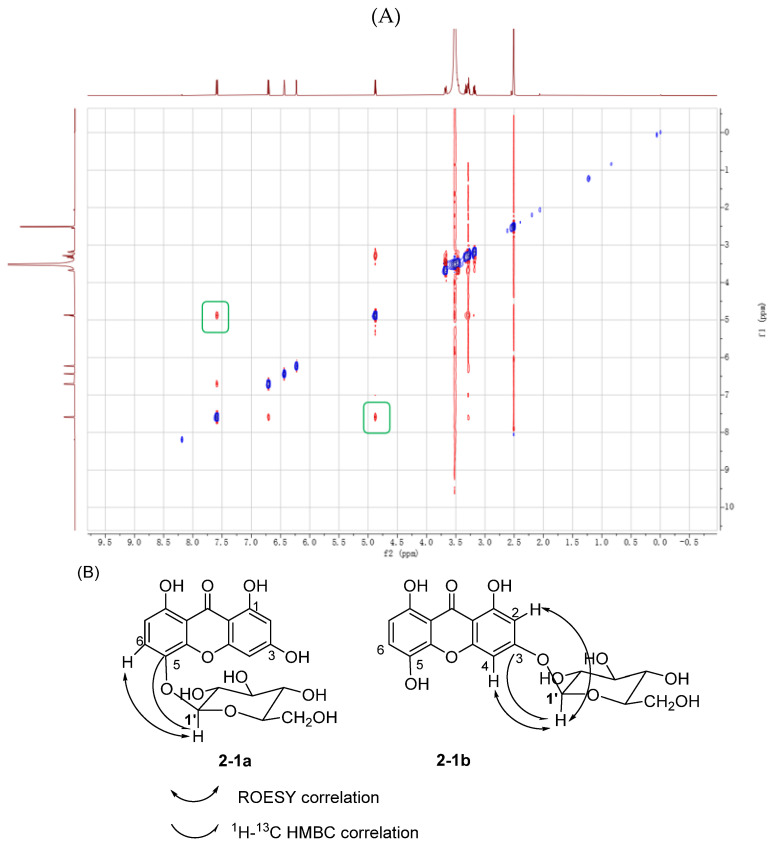
(**A**) ROESY spectrum of **2-1a** (DMSO-d_6_ + D_2_O, 600 MHz); The green boxes indicate the typical ^1^H-^1^H correlation of compound **2-1a**. (**B**) Key HMBC and ROESY correlations of the two 1,3,5,8-tetrahydroxyxanthone glucosides.

The structure of product **2-1a**, including its stereochemistry, was fully assigned using MS/MS, 1D and 2D NMR data. The MS/MS spectrum exhibited a loss of 162 amu (one glucose moiety), indicative of an *O*-glycosidic linkage (Supplementary Information Figure S29). The ^1^H NMR spectrum showed an anomeric proton signal of the β-D-glucopyranosy at δ 4.88 (H-1′, 1H, d, *J* = 7.6 Hz). Finally, the glycosylation site at *O*-5 was established by the characteristic HMBC correlation from δ 4.88 (H-1′) to δ 137.50 (C-5), and confirmed by a ROESY correlation between H-1′ and H-6 (δ 7.60) (Figure 3A,B). On the basis of the above mentioned evidence, the structure of **2-1a** was elucidated to be 1,3,5,8-tetrahydroxyxanthone 5-*O*-β-D-glucopyranoside.

### 2.3. A Comparative Analysis with UGT73AE1 Highlighted the Unique Regioselectivity of AbCGT

**Figure 4:**
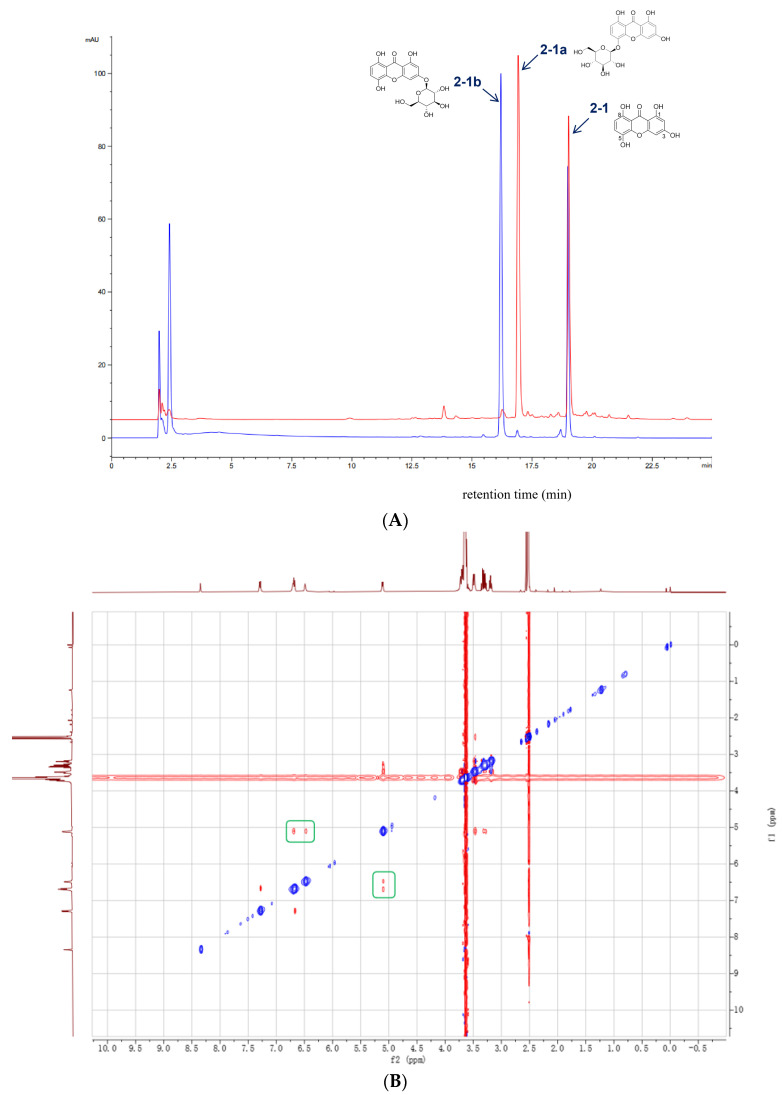
(**A**) HPLC chromatograms showing distinct product profiles of the two enzymes. AbCGT catalyzed the substrate **2-1** to yield product **2-1a** (red line), whereas UGT73AE1 catalyzed the same substrate **2-1** to produce **2-1b** (blue line). (**B**) ROESY spectrum of **2-1b** (DMSO-d_6_ + D_2_O, 500 MHz). The green boxes indicate the typical ^1^H-^1^H correlation of compound **2-1b**.

To gain deeper insights into the catalytic selectivity of AbCGT, we compared it with UGT73AE1, a homologous glycosyltransferase derived from safflower (*Carthamus tinctorius*). Upon employing demethylbellidifolin (**2-1**) as the substrate, UGT73AE1 catalyzed the formation of **2-1b** as the major product, with **2-1a** generated as a minor by-product in a ratio of 98.3:1.7. In contrast, AbCGT displayed a nearly reversed regioselectivity, affording **2-1a** and **2-1b** in a ratio of 97.5:2.5. HPLC analysis of the reaction mixtures directly illustrated the divergent regioselectivity of the two glycosyltransferases (Figure 4A). The MS/MS spectrum of **2-1b** confirmed an *O*-glucoside structure based on a characteristic loss of 162 amu (Figure S30). Ultimately, ROESY correlations between the anomeric proton H-1′ [δ 5.11, (1H, d, *J* = 7.5 Hz)] and H-2/H-4 (δ 6.47, δ 6.70) unambiguously identified **2-1b** as 1,3,5,8-tetrahydroxyxanthone 3-*O*-β-D-glucopyranoside (Figure 4B), demonstrating the high and contrasting site-specificity of the two enzymes.

**Figure 5:**
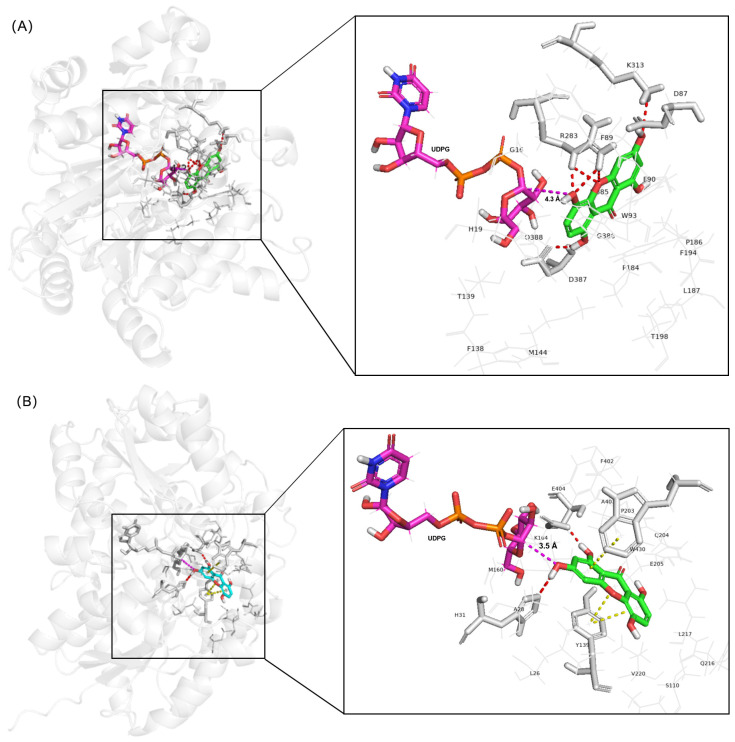
Molecular docking elucidates the structural basis for observed regioselective glycosylation of demethylbellidifolin (**2-1**) by AbCGT and UGT73AE1. (**A**) AbCGT active site with **2-1** and UDPG. The 5-OH group is oriented toward the anomeric carbon (C1) of UDPG (O5···C1 distance = 4.3 Å; pinkish purple dashed line), enabling nucleophilic attack. **2-1** forms eight hydrogen bonds with R283 (4 bonds), D387 (2 bonds), K313, and D87 (red dashed lines; 2.5–3.2 Å). (**B**) UGT73AE1 active site with **2-1** and UDPG. The 3-OH group is positioned for catalysis (O3···C1 distance = 3.5 Å; pinkish purple dashed line), stabilized by hydrogen bonds with H31 and E404, and π-π stacking with Y139 (2 interactions) and W430 (orange surfaces). Computational models generated with Schrodinger Glide (v2017.4); structures visualized in PyMOL v2.4.

Molecular docking analysis revealed distinct binding modes of demethylbellidifolin (**2-1**) in AbCGT and UGT73AE1 that rationalize their experimentally observed regioselectivities (Figure 5). In AbCGT, the 5-OH group of **2-1** is oriented toward the anomeric carbon (C1) of UDPG (O5···C1 distance = 4.3 Å), enabling nucleophilic attack; this pose is stabilized by eight hydrogen bonds with catalytic residues R283 (4 bonds), D387 (2 bonds), K313, and D87. Conversely, in UGT73AE1, the 3-OH group is positioned for catalysis (O3···C1 distance = 3.5 Å), with stabilization provided by hydrogen bonds (H31, E404) and π-stacking interactions (Y139, W430). These structural differences—particularly the proximity of specific hydroxyl groups to C1 and the distinct residue interaction networks—provide a molecular explanation for the exclusive 5-*O*- versus 3-*O*-glycosylation observed in product analysis. The dominant poses yielded docking scores of −6.121 kcal/mol (AbCGT) and −5.594 kcal/mol (UGT73AE1). Consistent with two-stage docking validation (Figure S6, Table S6), the observed regioselectivity arises from both optimal substrate positioning (Stage 1) and product stability (Stage 2).

### 2.4. Optimization of Enzymatic Conditions for Generation of Bioactive Product ***2-1a***

**Figure 6:**
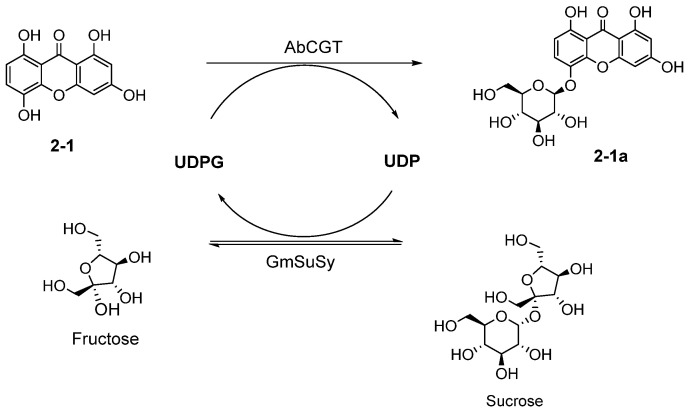
Scheme of the cascade reaction of AbCGT coupled with sucrose synthase GmSuSy used for regenerating UDPG.

To enable large-scale preparation of the active product **2-1a** for subsequent studies, we systematically optimized its enzymatic synthesis conditions. Due to the high cost of UDPG, we developed a dual-enzyme cascade system by coupling AbCGT with soybean-derived sucrose synthase (GmSuSy). This system utilizes low-cost sucrose and UDP as substrates to achieve in situ regeneration of UDPG, thereby significantly reducing production costs while driving the reaction equilibrium toward product formation (Figure 6).

**Figure 7:**
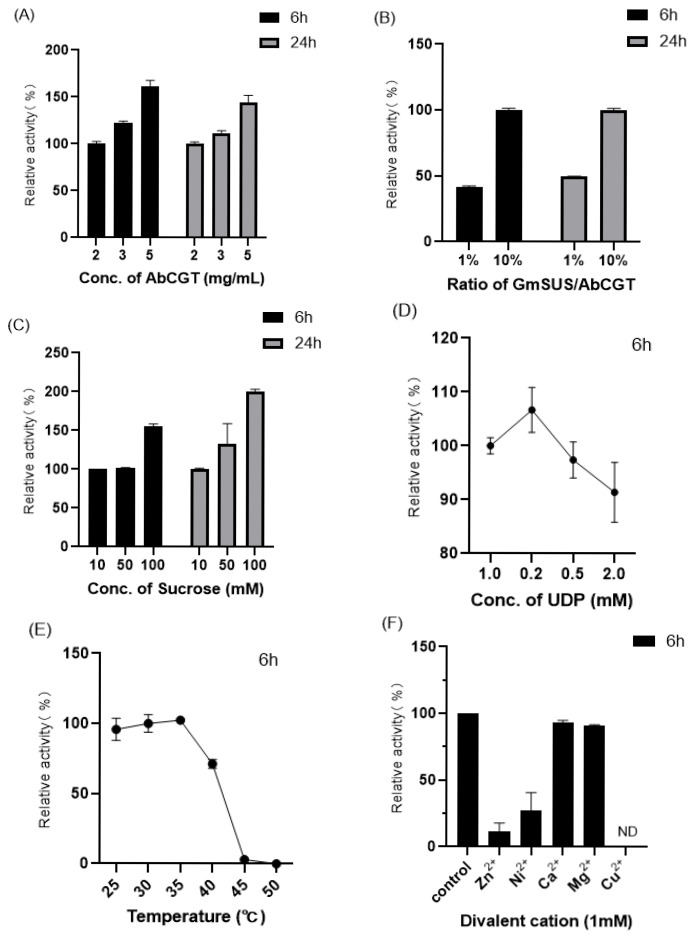
Optimization of the cascade reaction conditions for AbCGT-GmSuSy. After reaction for 6 hours or 24 h, the relative activity of enzymes was calculated by the production of **2-1a**. (**A**) Optimization of the concentration of AbCGT. (**B**) Optimization of the concentration ratio of GmSuSy/AbCGT. The concentration of AbCGT was 3 mg/mL. (**C**) Optimization of the concentration of sucrose. (**D**) Optimization of the concentration of UDP. (**E**) Thermal stability. (**F**) Optimization of divalent cation (with the final concentration of 1 mM). ND: not detected. The enzymatic activity was calculated by comparing the production of the glucoside **2-1a**. Error bars represent the standard deviation of three duplications.

The enzymatic activity was calculated by comparing the production of the glucoside **2-1a**. Firstly, the concentration of AbCGT was investigated. As expected, the production of xanthone glucoside increased as the enzyme concentration was raised from 2 mg/mL to 5 mg/mL(Figure 7A). We then optimized the ratio of GmSuSy to AbCGT. The yield of **2-1a** increased proportionally with elevated GmSuSy concentrations (Figure 7B), confirming that continuous UDPG regeneration was essential for driving the reaction equilibrium toward product formation and achieving high conversion. Based on these results, a combination of 3 mg/mL AbCGT and 10% (*w*/*w*) GmSuSy was selected for the subsequent cascade reaction.

Since the supply of sugar donor UDPG significantly affected the glycosylation yield, the concentrations of sucrose and UDP were optimized. Increasing sucrose concentration from 10 mM to 100 mM progressively enhanced the production of **2-1a** (Figure 7C). In contrast, higher UDP concentrations (0.2–2 equivalents) slightly suppressed the yield (Figure 7D). Based on these findings, the optimal reaction conditions were established as 100 mM sucrose with 0.2 equivalents of UDP.

The catalytic performance of the dual-enzyme system was found to be temperature-dependent, with optimal activity observed between 25–35 °C (Figure 7E). A significant reduction in enzymatic activity occurred while temperatures exceeding 35 °C, reflecting the system’s limited thermal stability.

The screening of various divalent metal ions revealed different inhibitory effects on the enzymatic reaction (Figure 7F). Notably, Ni^2+^ and Zn^2+^ demonstrated the most obvious inhibition of catalytic efficiency, while Mg^2+^ and Ca^2+^ showed a slight impact on enzyme activity. Cu^2+^ was hypothesized to oxidize substrate **2-1**, since the complete absence of both substrate and glycosylation product was observed.

Based on these findings, the optimized reaction mixture consisted of 50 mM PBS (pH 7.4), 0.2 mM UDP, 100 mM sucrose, 3 mg/mL AbCGT and GmSuSy (10% of AbCGT in weight), without addition of any metal cation.

### 2.5. Targeted Mutagenesis of AbCGT to Enhance Catalytic Activity for ***2-1a*** Synthesis

To improve the synthesis efficiency of target product **2-1a**, we performed residue-focused mutagenesis on AbCGT guided by molecular docking analysis. Although the crystal structure of the AbCGT-substrate **2-1** complex was unavailable, we employed molecular docking simulations to predict key amino acid residues in its active pocket. The docking results (Figure 5A) revealed that residues H19, F90, W93, F138, F194, G386, P186, L187, and T198 collectively form the binding pocket for substrate **2-1**. UDPG binding site appeared to involve residues S282, H363, N367, and S368 for phosphate group coordination (Supplementary Information Figures S5 and S6).

**Figure 8:**
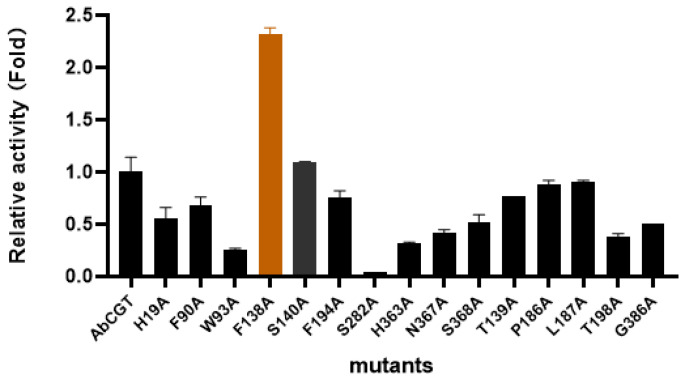
Relative catalytic activity of 15 mutants compared to AbCGT wild type. The mutant with the highest catalytic activity was colored in orange.

Based on these findings, we performed systematic alanine scanning mutagenesis of the predicted key residues. The enzymatic activity was calculated by comparing the production of the glucoside **2-1a**. Under optimized reaction conditions, enzymatic activity assays revealed that the F138A mutant exhibited significantly enhanced catalytic performance, demonstrating a 2.3-fold increase in activity compared to wild-type AbCGT (Figure 8). While all the other mutants negatively affected the catalytic activity. Kinetic parameter analysis (Table 1) further confirmed these improvements, showing substantially elevated Vmax and Km values for the mutant enzyme F138A.

## 3. Materials and Methods

### 3.1. Plasmids and Chemicals

The strains and plasmids used in this study are listed in Supplementary Table S1. AbCGT from *Aloe barbadensis* (accession numbers: MN747045) sucrose synthase from *Glycine max* (GmSuSy) (accession number: NP_001237525) and UGT73AE1 from *Carthamus tinctorius* (accession numbers: KJ956788) were codon optimized and synthesized by GenScript (Nanjing, China). *Escherichia coli* (*E. coli*) Top10 was used for plasmid construction. pET-28a (+) and *E. coli* BL21 (DE3) were employed for heterologous expression of AbCGT (including its variants) and UGT73AE1. The gene of AbCGT was inserted into the pET-28a (+) under the T7 promoter using a seamless cloning and assembly kits from Vazyme (Nanjing, China). Site-directed mutagenesis of AbCGT was performed by PCR amplification with primers listed in Supplementary Table S2. All gene insertions and mutations were verified by Sanger sequencing (Tsingke, Shanghai, China). The substrates **1-9** and **1-11** were from our compound library. Other substrates were purchased from Yuanye Bio-Technology (Shanghai, China). All other chemical reagents were purchased from Sangon Biotech (Shanghai, China).

### 3.2. Expression and Purification of AbCGT, GmSuSy and UGT73AE1

Plasmids with target genes were transformed into *Escherichia coli* BL21 (DE3). A 10 mL overnight pre-culture of *E. coli* BL21 (DE3) in LB medium was inoculated into a 1 L of fresh LB medium (in a 2 L Erlenmeyer flask) supplemented with kanamycin (100 μg/mL). The culture was incubated at 37 °C with shaking (200 rpm) until reaching an OD600 of 0.6–0.8. The recombinant protein expression was achieved by induction with 0.1 mM Isopropyl β-D-thiogalactoside (IPTG) at 18 °C for 12 h with shaking (200 rpm). The cells were then harvested by centrifugation (4000× *g*, 10 min, 4 °C) and resuspended in ice-cold binding buffer (500 mM NaCl, 50 mM phosphate buffer, pH 7.4) containing 1 mM phenylmethylsulfonyl fluoride (PMSF). Cell disruption was performed using a high-pressure homogenizer (Union-Biotech, Shanghai, China). Cell debris was immediately removed by centrifugation at 10,000× *g* for 60 min at 4 °C. The soluble fraction was passed through a 0.22 μm membrane and applied to 1 mL Ni-NTA resin loaded in a column, which was pre-equilibrated with binding buffer.

After washing with 10 column volumes of binding buffer (flow rate: 1 mL/min, 4 °C), the target protein was eluted using a linear imidazole gradient (500 mM NaCl, 50–200 mM imidazole, and 50 mM PBS, pH 7.4). All fractions were analyzed using SDS-PAGE, and protein concentration was determined using the Bradford protein assay (Sangon Biotech). Finally, the recombinant protein was desalted by the desalting column and concentrated using a spin concentrator (30 kDa MWCO, 15 mL, Amicon Ultra, Merck Millipore Ltd., Darmstadt, Germany). The protein solutions were immediately used for enzymatic assays or stored at −80 °C.

### 3.3. Enzymatic Activity Determination

To determine the catalytic activity of AbCGT, 3 mg/mL enzyme was incubated with 1 mM demethylbellidifolin (compound **2-1**) in a 100 μL reaction system containing 2 mM UDPG and 50 mM PBS pH 7.4 at 30 °C for 2 h. Then, the reaction was quenched by adding equal volume of methanol. Samples were prepared by centrifugation at 15,000× *g* for 8 min and filtered with 0.22 μm filters.

HPLC separation was achieved using an Agilent (Agilent Technologies Inc. Palo Alto, CA, USA) 1200 system equipped with an Agilent ZORBAX SB-C18 column (4.6 × 150 mm, 3.5 μm) maintained at 25 °C, with mobile phases consisting of 0.1% (*v*/*v*) formic acid in water (solvent A) and HPLC-grade methanol (solvent B). The flow rate was maintained at 0.70 mL/min with detection at 254 nm using a variable wavelength detector. Quantification was performed against an external standard curve of substrate **2-1**, with all experiments conducted in triplicate (data presented as mean ± SD). For structural confirmation, LC-MS analysis was performed in negative electrospray ionization mode on an Agilent single quadrupole mass spectrometer coupled to a ZORBAX Extend-C18 column (4.6 × 150 mm, 3.5 μm), using a methanol/water (0.1% formic acid) mobile phase system. The complete HPLC gradient profile and standard curve data are provided in the Supplementary Information Table S3, Figure S8.

### 3.4. Scale-Up Enzymatic Reactions

For scale-up enzymatic reactions typically 10–30 μM of aglycone was initially dissolved in 200 μL DMSO and subsequently diluted with 50 mM phosphate-buffered saline (PBS, pH 7.4) to a final volume of 15 mL. For the substrates in Figure 1 (from **1-1** to **1-12**, and **2-1**), the reaction system was supplemented with 20–60 μM UDPG and 20–40 mg of purified AbCGT enzyme. The yield was listed in Supplementary Information Table S4. For the improved scaled-up reaction of substrate **2-1**, the reaction system was supplemented with 10–30 μmol UDP and 30–45 mg of purified AbCGT enzyme (or UGT73AE1) and GmSuSy (10%, *w*/*w*), and sugar donor (100 mM). The scaled-up reactions were carried out at 30 °C for 24 h with constant agitation.

After completion, the reaction mixtures were immediately frozen at −80 °C and subsequently lyophilized to complete dryness using a FreeZone freeze dryer (Labconco, Kansas City, MO, USA). The resulting residue was reconstituted in 2.0 mL HPLC-grade methanol and subjected to purification via reverse-phase semi-preparative HPLC (C18 column, 250 × 10 mm, 5 μm particle size). The purified compounds were characterized by high-resolution mass spectrometry (HRMS) or LC-MS. Main products were confirmed by comprehensive NMR analysis (^1^H, ^13^C, and ROESY).

### 3.5. Optimization of the AbCGT-GmSuSy Cascade Reaction

To enhance the synthesis of **2-1a** from substrate **2-1**, a cascade reaction system coupling AbCGT with sucrose synthase GmSuSy was established. Key parameters, including enzyme ratio, sucrose and UDP concentrations, and temperature, were systematically optimized. The standard reaction mixture (100 μL total volume) consisted of 50 mM PBS (pH 7.4), 2 mM **2-1**, 1 mM UDP, 100 mM sucrose, and 3 mg/mL AbCGT and GmSuSy (10% *w*/*w* relative to AbCGT). Reactions were conducted at 30 °C for 6 h, terminated by adding an equal volume of methanol, and centrifuged (15,000× *g*, 8 min). The resulting supernatants were analyzed as previously described.

To determine optimal conditions, the effects of enzyme AbCGT concentration (2–5 mg/mL), enzyme ratio (AbCGT:GmSuSy), sucrose concentration (10–100 mM), and UDP concentration (0.2–2 mM) were investigated. Additionally, thermostability was assessed by pre-incubating the enzyme mixture (10% GmSuSy relative to AbCGT) at different temperatures (25–50 °C, 30 min, pH 7.4) before initiating the reaction with 1 mM **2-1**. Relative enzyme activity was calculated based on HPLC peak areas of glycosylated products and substrates. All experiments were performed in triplicate, with data presented as mean ± standard deviation (SD).

### 3.6. Effects of Divalent Metal Ions on Enzymatic Activity

To evaluate the influence of divalent metal ions on the AbCGT-GmSuSy cascade system, enzymatic reactions were conducted in the presence of CaCl_2_, CuCl_2_, NiCl_2_, MgCl_2_, and ZnCl_2_ (each at 1 mM final concentration). The standard reaction mixture (100 μL total volume) contained 1 mM **2-1**, 100 mM sucrose, 3 mg/mL purified AbCGT, and 0.3 mg/mL purified GmSuSy in 50 mM PBS (pH 7.4). Reactions were incubated at 30 °C for 6 h, and enzyme activity was quantified by HPLC analysis of product formation. All assays were performed in triplicate, with data expressed as mean ± standard deviation (SD).

### 3.7. Enzyme Kinetics of AbCGT and AbCGT-F138A

The kinetic parameters (Km, Vmax, and catalytic efficiency) of both wild-type AbCGT and its variant AbCGT-F138A were determined using substrate **2-1** at varying concentrations (0.2–2.5 mM). Reactions were performed in 50 mM PBS (pH 7.4) at 30 °C for 2 h, terminated by adding an equal volume of methanol, and centrifuged (15,000× *g*, 8 min). Kinetic analysis was conducted using Lineweaver–Burk plots, with all experiments performed in triplicate to ensure statistical reliability.

### 3.8. Molecular Docking Methods

Homology Modeling: Using the relevant amino acid sequence, structural models of AbCGT and UGT73AE1 were constructed via the SWISS-MODEL server, with the crystal structure 6l5r (SMTL ID) serving as the template. Only chain A was used to perform later calculation.

Protein Preparation: Receptor structures were prepared using the Protein Preparation Wizard workflow implemented in the Maestro module of the Schrödinger software package v2017.4 (Schrödinger, Inc., New York, NY, USA). The default protocol was employed, which includes removal of water molecules beyond 5 Å from the ligand, addition of missing hydrogen atoms, assignment of protonation states and partial charges, and restrained minimization with a root-mean-square deviation (RMSD) cutoff of 0.3 Å for hydrogens only. Receptor grid for docking was subsequently generated using the Glide module in Schrödinger. The grid box was defined as a cubic region of 12 × 12 × 12 Å centered on the co-crystallized ligand (ML-349) in the complex structure.

Ligand Preparation: Ligands were prepared using LigPrep to generate stereoisomers and tautomers. Protonation states at pH 7.0 ± 2.0 were predicted using Epik. All other parameters were set to their default values.

Molecular Docking: Ligand docking was performed using the Ligand Docking module in Schrödinger. The receptor grid files and ligand structures generated from the protein and ligand preparation steps were used as input. Docking was conducted using the standard precision (SP) mode, and up to four top-ranked poses were retained for each ligand. All other docking parameters were maintained at their default settings.

### 3.9. Cell Culture and Treatment

Normal rat kidney interstitial fibroblasts (NRK-49F) were maintained in Dulbecco’s Modified Eagle Medium (DMEM; D6429, Sigma-Aldrich, Saint Louis, MO, USA) supplemented with 10% fetal bovine serum (FBS) at 37 °C. The cells that reached approximately 50% confluence were used for in vitro experiments. The cells were serum-starved for 12 h and were pretreated with compound for 1 h prior to incubation with TGF-β1 (10804-HNAC, Sino Biological, Beijing, China) at 4 ng/mL. Cells were collected at 24 h after TGF-β1 treatment, respectively. Whole cell lysates were prepared and subjected to Western blot analyses [36].

### 3.10. Western Blot Analysis

Western blot analysis was performed as previously described [37]. The primary antibodies used were as follows: anti-fibronectin (Ab2413; Abcam, Cambridge, UK), anti-α-SMA (19245S; CST), and tubulin (2144S; CST). For quantification, the protein bands were analyzed with ImageJ software 1.50i (National Institutes of Health, Bethesda, MD, USA).

## 4. Conclusions

This study established a systematic glycosylation method for phenolic compounds using the glycosyltransferase AbCGT. The resulting 1,3,5,8-tetrahydroxyxanthone 5-*O*-β-D-glucopyranoside (**2-1a**), synthesized from demethylbellidifolin (compound **2-1**), demonstrated significant anti-renal fibrotic activity. In contrast to AbCGT, the homologous glycosyltransferase UGT73AE1 glycosylated the substrate at the 3-OH position. Molecular docking analysis rationalized the distinct regioselectivity between AbCGT (5-*O*-glycosylation) and its homolog UGT73AE1 (3-*O*-glycosylation), highlighting the critical role of active-site architecture. To optimize the production of **2-1a**, we constructed a UDPG recycling system by coupling AbCGT with *Glycine max* sucrose synthase (GmSuSy). Furthermore, alanine scanning of residues highlighted by docking studies identified position F138 as critical; the F138A mutation subsequently enhanced the catalytic efficiency of AbCGT by 2.3-fold. Our work provides not only a promising xanthone-based scaffold glucoside for anti-renal fibrosis drug development but also an efficient biosynthetic strategy and engineering framework for glycosyltransferases.

## Figures and Tables

**Table 1:** Kinetic parameters of wild-type AbCGT and F138A mutant.

	Vmax	Km
AbCGT (wild-type)	0.0111	1.239
F138A	0.0520	5.670

## Data Availability

Data are contained within the article and Supplementary Materials.

## Supplementary Materials

The following supporting information can be downloaded at: https://www.mdpi.com/article/10.3390/molecules31020309/s1, Figure S1: SDS-PAGE analysis of purified recombinant His10-AbCGT; Figure S2: Fragmentation of C-hexosides under collision-induced dissociation tandem mass spectrometry (CID-MS/MS) to generate fragment losses of 90 and 120 amus; Figure S3: Western blot of glycosides **1-1a~1-12a** and **2-1a**; Figure S4: Western blot of demethylbellidifolin (**2-1**) and its glycoside **2-1a**; Figure S5: Two-dimensional ligand interaction networks in the AbCGT ternary complex (**2-1**/UDPG/AbCGT) derived from molecular docking; Figure S6: Two-stage molecular docking validates regioselectivity of AbCGT and UGT73AE1 through substrate positioning and product stability analysis; Figure S7: Enzyme kinetic study; Figure S8: Standard curve of substrate **2-1**; Figures S9–S17: NMR spectrum of products; Figures S18–S30: Typical MS^2^ spectrum of glycosylated products; Table S1: The strains and plasmids used in this study; Table S2: Primers used in this study; Table S3: HPLC method used in this study; Table S4: The yield of glycosides in the scale-up reactions; Table S5: The purity of glycosides in Figure 2A by HPLC; Table S6: Post-glycosylation docking scores of all possible glycosylated products with glycosyltransferases and UDP cofactor.
