# Supplementary material for: Regioselective Glycosylation of Demethylbellidifolin by Glycosyltransferase AbCGT Yields Potent Anti-Renal Fibrosis Compound
Source: Molecules. 2026 Jan 15;31(2):309. doi: 10.3390/molecules31020309 (PMC12844164; doi:10.3390/molecules31020309)
Supplement: Supplementary file 1 [file molecules-31-00309-s001.zip › molecules-4053715-supplementary.pdf]

# Supplementary Information

## Regioselective Glycosylation of Demethylbellidifolin by Glycosyltransferase AbCGT Yields Potent Anti-renal Fibrosis Compound

**Limin Zeng[12]†, Shichao Cui[2]†, Xingyu Ji[2]†, Yuhong Liu[2], Guozhang Long[23], Yulan Xia[23], Gang Cheng[4*], Jingya Li[1235*], Youhong Hu[1235*]**

1, School of Chinese Materia Medica, College of Pharmacy, Nanjing University of Chinese Medicine, Nanjing 210023, China;

2, State Key Laboratory of Drug Research, Shanghai Institute of Materia Medica, Chinese Academy of Sciences, Shanghai 201203, China;

3, University of Chinese Academy of Sciences, Beijing 100049, China;

4, School of Pharmaceutical Sciences, Zhejiang Chinese Medical University, Hangzhou 310053, China;

5, Key Laboratory of Glyco-drug Research of Zhejiang Province, School of Pharmaceutical Science and Technology, Hangzhou Institute for Advanced Study, University of Chinese Academy of Sciences, Hangzhou 310024, China;

[*] Correspondence: E-mail: yhhu@simm.ac.cn (Y.H.); jyli@simm.ac.cn (J.L.); gangcheng@zcmu.edu.cn (G.C.).

† These authors contributed equally to this work.

## Contents

1. General information...

2. Characterization data of products...

3. Copies of $^1$H NMR, ROESY, and $^{13}$C NMR spectra.

4. Copies of MS-MS spectrum..

## 1. General information

Table S1 The strains and plasmids used in this study

| Strains or Plasmids | Description | Sources |
|---|---|---|
| **Strains** | | |
| Top10 | wild type | WeidiBio |
| BL21(DE3) | wild type | WeidiBio |
| BL21-*AbCGT* | *E. coli* BL21(DE3) harboring plasmid pET-28a(+)-*AbCGT* | this study |
| BL21-UGT73AE1 | *E. coli* BL21(DE3) harboring plasmid pET-28a(+)-UGT73AE1 | this study |
| BL21-*Gm*SuSy | *E. coli* BL21(DE3) harboring plasmid pET-28a(+)-*Gm*SuSy | this study |
| **Plasmids** | | |
| pET28a-*AbCGT* | pET-28a(+) carry a glycosyltransferase *AbCGT* gene from *Aloe barbadensis* | GenScript |
| pET28a-UGT73AE1 | pET-28a(+) carry a glycosyltransferase *UGT73AE1* gene from *Carthamus tinctorius* | GenScript |
| pET28a-*Gm*SuSy | pET-28a(+) carry a sucrose synthase *Gm*SuSy gene from *Glycine max* (soybean) | GenScript |

Table S2 Primers used in this study

| Primer name[a] | Sequence (5' to 3') |
|---|---|
| H19A-F | GCGCTGACCCCGTTTTGTCGTCTGGC |
| H19A-R | CGGGGTCAGCGCACCCATACCCGCGCTCGG |
| F90A-F | GCGCAACAATGGGAAAGCATCCGTCGTTGC |
| F90A-R | CCATTGTTGCGCGAACGGATCCGGGCTCGACGGGAACTG |
| W93A-F | GCGGAAAGCATCCGTCGTTGCGCACACTT |
| W93A-R | GATGCTTTCCGCTTGTTGGAAGAACGGATCCGGGCTCGACGG |
| F138A-F | GCGACCAGCAGTGCCAGCATGC |
| F138A-R | ACTGCTGGTCGCTAAGATGTATGCCGGGATGTTCAGCTGC |
| S140A-F | GCGAGTGCCAGCATGCTGTCTCTG |
| S140A-R | GCTGGCACTCGCGGTGAATAAGATGTATGCCGGGATGTT |
| F194A-F | GCGTCCACGCTGACGGTTGATAATGGTCGTTGC |
| F194A-R | ATTATCAACCGTCAGCGTGGACGCGATATGACCCGGAACATGCAG |
| S282A-F | GCGCGTACCGCTATGTCCCCGGAG |
| S282A-R | AGCGGTACGCGCGCCGAAGCTCACGTACACCAC |
| H363A-F | GCGTGCGGTTGGAACAGCGTTACCGAA |
| H363A-R | CCAACCGCACGCGGAAACAAAGCCGCCAACAGC |
| N367A-F | GCGAGCGTTACCGAAGCAGCGGTC |
| N367A-R | GGTAACGCTCGCCCAACCGCAGTGGGAAACAAAGCC |
| S368A-F | GCGGTTACCGAAGCAGCGGTCGCGGGT |
| S368A-R | TTCGGTAACCGCGTTCCAACCGCAGTGGGAAACAAAGCCGCC |
| T139A-F | CTTATTCCGCAGCAGTGCCAGCATGCTGTCTC |
| T139A-R | CACTGCTGCGGAATAAGATGTATGCCGGGATGT |
| P186A-F | TTCCACCACGCCTGCATGTTCCGGGTCATATC |
| P186A-R | ATGCAGGCGTGGTGGAACCCAGGCTGCCGGAA |
| L187A-F | ACCACCGCGCCATGTTCCGGGTCATATCTTTTC |
| L187A-R | GAACATGGCGCGGTGGTGGAACCCAGGCTGCC |
| T198A-F | ACGCTGCGCGTTGATAATGGTCGTTGCCTGCC |
| T198A-R | TTATCAACGCGCAGCGTGGAAAAGATATGACCC |
| G386A-F | ACGTATGCGCGACCAACGTCTGAATGCAGAAGT |
| G386A-R | GTTGGTCGCGCATACGTGGCCAACATAGCACA |

a: H: Histidine (His); A: alanine (Ala); F: phenylalanine (Phe); W: Tryptophan (Trp); F: Phenylalanine (Phe); S: Serine (Ser); N: Asparagine (Asn); T: threonine (Thr); P: Proline (Pro); L: leucine (Leu); G: glycine (Gly).

Table S3 HPLC method used in this study

| t/min | A (water [0.1% FA] /% | B (methanol)/% |
|---|---|---|
| 0.0 | 70.0 | 30.0 |
| 3.0 | 70.0 | 30.0 |
| 12.0 | 30.0 | 70.0 |
| 15.0 | 10.0 | 90.0 |
| 20.0 | 10.0 | 90.0 |
| 22.0 | 70.0 | 30.0 |
| 25.0 | 70.0 | 30.0 |

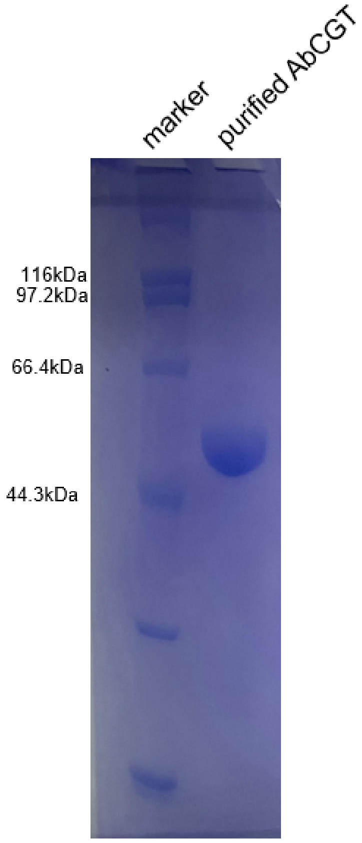


Figure S1 SDS-PAGE analysis of purified recombinant His10-AbCGT (52.4kDa)

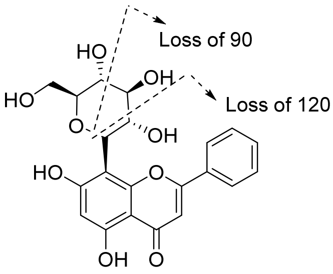


Figure S2 Fragmentation of C-hexosides under collision-induced dissociation tandem mass spectrometry (CID-MS/MS) to generate fragment losses of 90 and 120 amus

Table S4 The yield of glycosides in the scale-up reactions

| substrate | product (glycoside) | yield (%) |
|---|---|---|
| **1-1** | **1-1a** | 45.8 |
| **1-2** | **1-2a** | 14.0 |
| **1-3** | **1-3a** | 36.7 |
| **1-4** | **1-4a** | 45.1 |
| **1-5** | **1-5a** | 10.1 |
| **1-6** | **1-6a** | 26.1 |
| **1-7** | **1-7a** | 38.1 |
| **1-8** | **1-8a** | 8.6 |
| **1-9** | **1-9a** | 11.6 |
| **1-10** | **1-10a** | 15.3 |
| **1-11** | **1-11a** | 18.7 |
| **1-12** | **1-12a** | 37.4 |
| **2-1** | **2-1a** | 18.9 |
| **2-1** | **2-1b** | 28.4 |
| **2-1 *** | **2-1a** | 44.7 |

2-1 *: the yield of 2-1a using the optimized condition.

Table S5 The purity of glycosides in Figure 2A by HPLC

| Product (glycoside) | Purity |
|---|---|
| **1-1a** | >99% |
| **1-2a** | >99% |
| **1-3a** | 82.9% |
| **1-4a** | >99% |
| **1-5a** | 78.7% |
| **1-6a** | >99% |
| **1-7a** | 79% |
| **1-8a** | >99% |

| | |
|---|---|
| **1-9a** | >99% |
| **1-10a** | >99% |
| **1-11a** | >99% |
| **1-12a** | 81.4% |

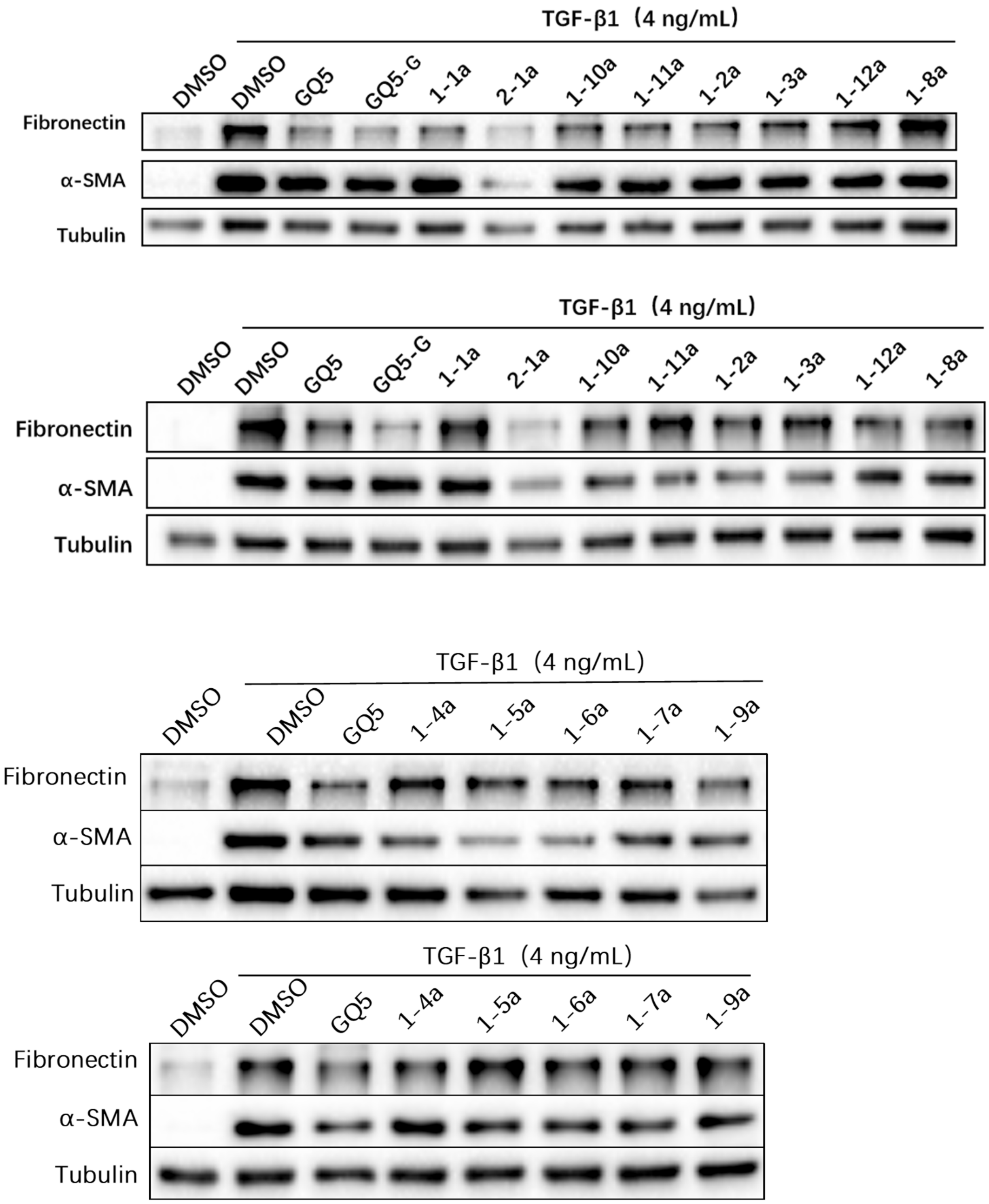


Figure S3 Western blot of glycosides **1-1a**~**1-12a** and **2-1a**. NRK49F cells were preincubated with the glycosides (5 μM) for 1 hour before TGF-β1 (4 ng/ml) treatment. Cells were harvested 24 hours after TGF-β1 stimulation. Whole cell lysates were

prepared and subjected to Western blot analyses. GQ5 was a natural phenolic compound that was reported to demonstrate the capacity of inhibiting protein expression of fibronectin and α-smooth muscle actin (α-SMA). GQ5-G was the glucoside of GQ5. Both compounds were applied in the assay to evaluate the anti-renal fibrosis effect.

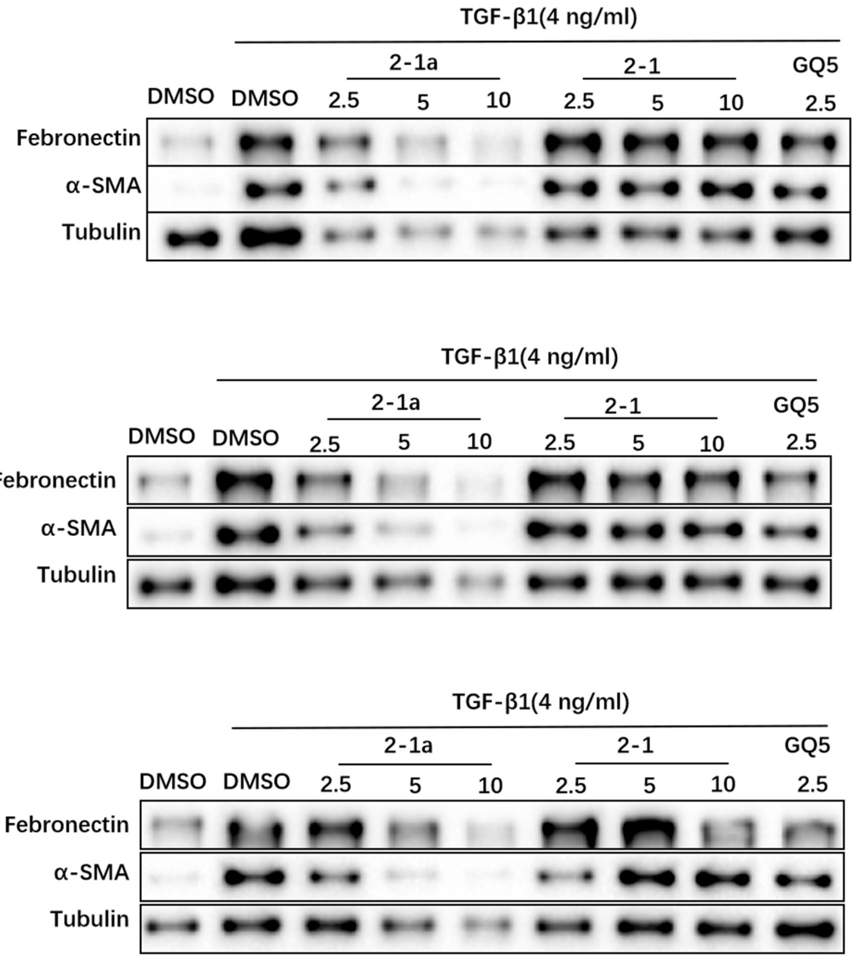


Figure S4 Western blot of demethylbellidifolin (**2-1**) and its glycoside **2-1a**. NRK49F cells were preincubated with the compounds (2.5 μM, 5 μM, 10 μM) for 1 hour before TGF-β1 (4 ng/ml) treatment. The concentration of GQ5 was 2.5 μM. The cells were treated as previously described.

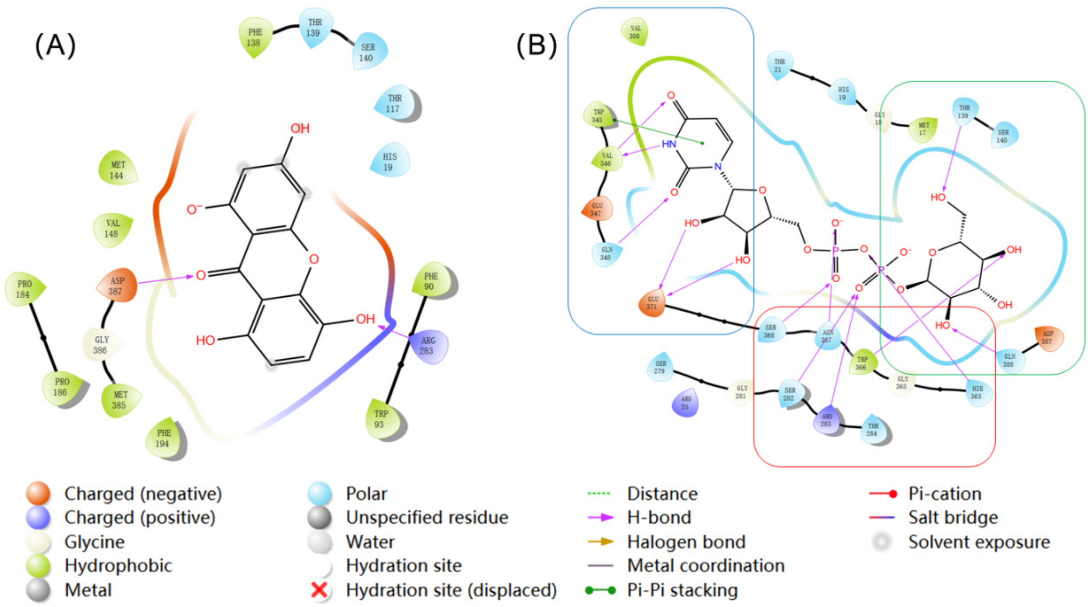


Figure S5. Two-dimensional ligand interaction networks in the AbCGT ternary complex (**2-1**/UDPG/AbCGT) derived from molecular docking. (A) **2-1** interactions: Hydrogen bonds (pinkish purple arows; ≤3.5 Å) with R283, D387; hydrophobic contacts (pink arcs; ≤4.0 Å) with F138, F194, W93 etc. (B) UDPG interactions.

Table S6. Post-glycosylation docking scores of all possible glycosylated products with glycosyltransferases and UDP cofactor

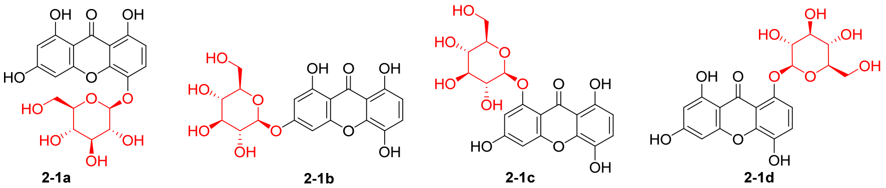


| **glycoside** | Glycosylation site | glycosyltransferase | Docking score |
|---|---|---|---|
| **2-1a** | 5-*O*-glucoside | AbCGT | **-9.55** |
| **2-1b** | 3-*O*-glucoside | AbCGT | -6.96- |
| **2-1c** | 1-*O*-glucoside | AbCGT | -7.56 |
| **2-1d** | 8-*O*-glucoside | AbCGT | -7.90 |
| **2-1a** | 5-*O*-glucoside | UGT73AE1 | -7.58 |
| **2-1b** | 3-*O*-glucoside | UGT73AE1 | **-7.89** |
| **2-1c** | 1-*O*-glucoside | UGT73AE1 | -7.54 |

| | | | |
|---|---|---|---|
| **2-1d** | 8-*O*-glucoside | UGT73AE1 | -7.32 |

Note: Lowest energy poses (bold and red) correspond to experimentally observed regioselectivity. Values represent triplicate docking runs (SD < 0.3 kcal/mol).

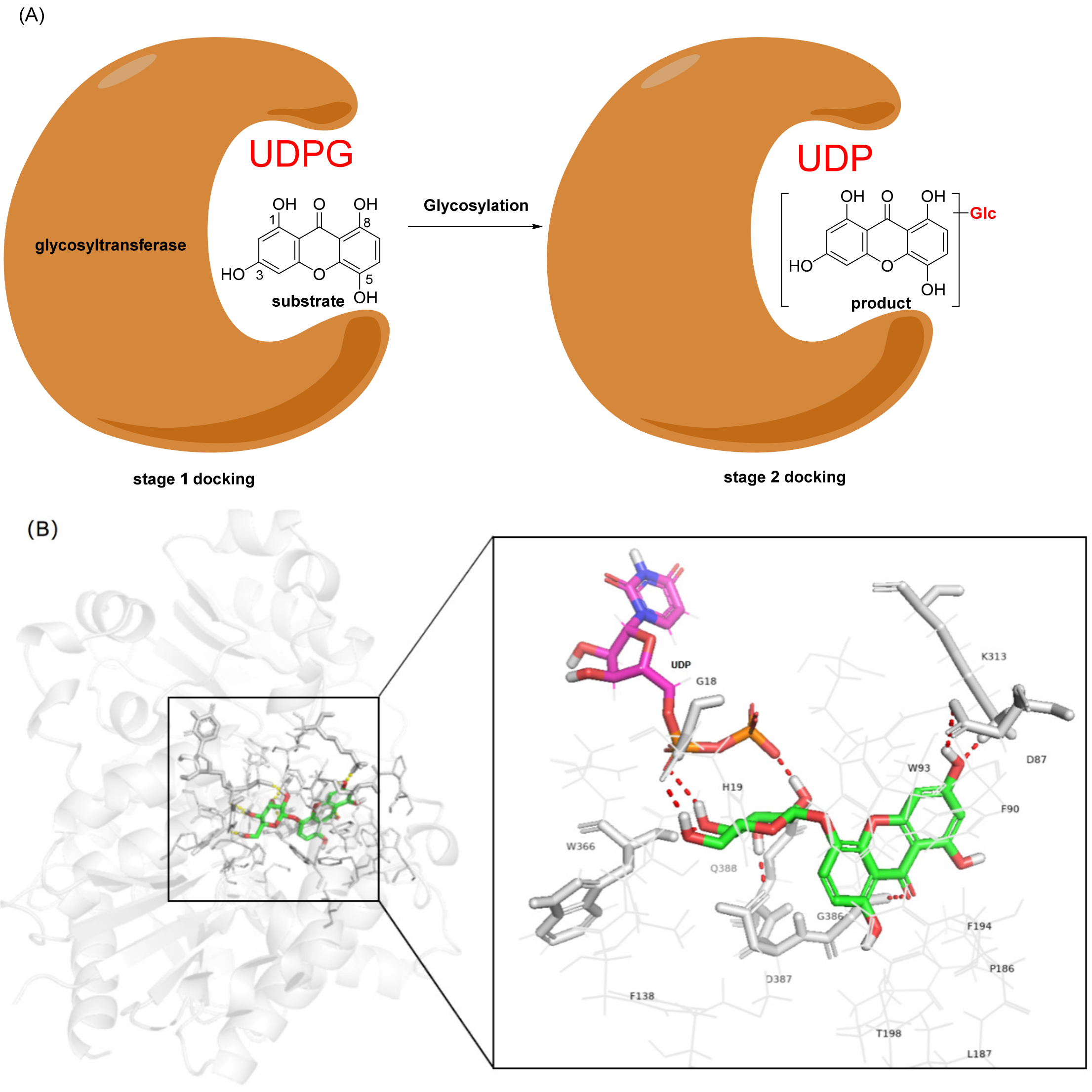

(C)

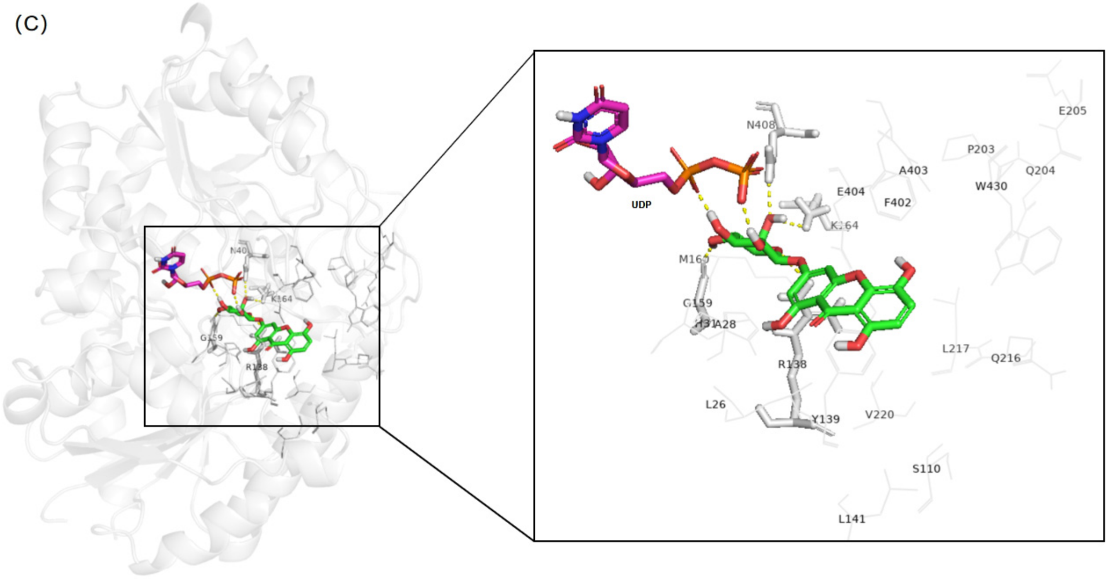


**Figure S6**. Two-stage molecular docking validates regioselectivity of AbCGT and UGT73AE1 through substrate positioning and product stability analysis.

(A) Strategy schematic: Stage 1 (pre-glycosylation) identifies catalytically competent hydroxyl orientation (poses in Figure 5); Stage 2 (post-glycosylation) evaluates binding stability of all four possible glycosylated products (**2-1a - d**) with enzyme-UDP complexes.

(B) AbCGT system: The 5-*O*-glucoside (**2-1a**) exhibits strongest binding (−9.55 kcal/mol; Table S6), forming 8 hydrogen bonds distributed across glucose (UDP × 2, Q388 × 2, W366, D387) and aglycone moieties (G386, K313, D87). This dual stabilization rationalizes exclusive 5-*O*-glycosylation.

(C) UGT73AE1 system: The 3-*O*-glucoside (**2-1b**) shows optimal binding (−7.89 kcal/mol; Table S6), with 5 hydrogen bonds exclusively via glucose (UDP × 2, K164, N408, R138). The solvent-exposed aglycone (right panel) suggests facile product release. Other isomers show >0.3 kcal/mol weaker binding (Table S6), confirming strict 3-*O*-preference. Docking performed with with Schrödinger Glide (v2017.4); structures visualized in PyMOL v2.4. Aglycone colored orange; hydrogen bonds: blue dashed lines (2.7 – 3.2 Å).

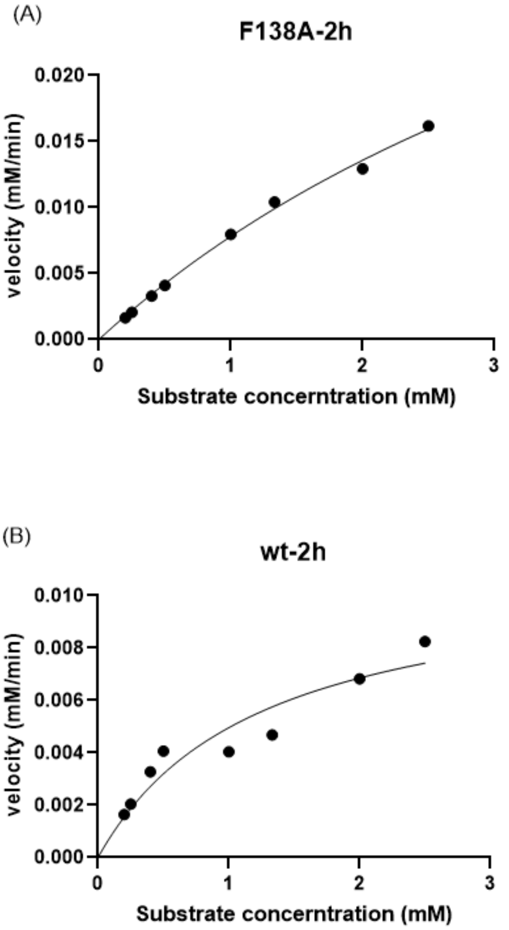


Figure S7 (A) Enzyme kinetic study of the mutant F138A after reaction for 2 hours; (B)Enzyme kinetic study of AbCGT wild-type after reaction for 2 hours.

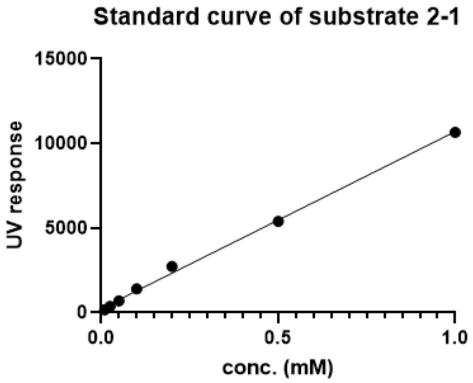


Figure S8 Standard curve of substrate **2-1**

## 2. Characterization data of products

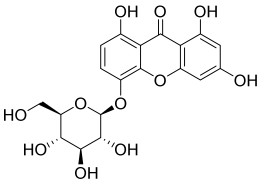


2-1a

### 5-*O*-β-D-glucosyl-1,3,8-trihydroxyxanthone(2-1a)

**$^1$H NMR (DMSO-d$_6$+D$_2$O [10:1], 600 MHz):** δ 7.60 (d, *J* = 9.1 Hz, 1H), 6.72 (d, *J* = 9.0 Hz, 1H), 6.45 (d, *J* = 2.1 Hz, 1H), 6.24 (d, *J* = 2.1 Hz, 1H), 4.88 (d, *J* = 7.6 Hz, 1H), 3.69 (dd, *J* = 12.0, 2.1 Hz, 1H), 3.47 (d, *J* = 5.7 Hz, 1H), 3.34 (dd, *J* = 9.1, 7.6 Hz, 1H), 3.30 (dq, *J* = 11.3, 4.4 Hz, 2H), 3.21 – 3.17 (m, 1H). Parameters: Pulse sequence = zg30, Number of scans = 12, Relaxation delay = 1s. **$^{13}$C NMR (126 MHz, DMSO-d$_6$):** δ 183.64, 167.37, 162.35, 157.88, 154.64, 145.73, 137.53, 126.04, 109.63, 107.71, 102.46, 101.51, 99.17, 95.24, 77.47, 76.77, 73.62, 70.01, 61.05. Pulse sequence = zgpg30, Number of scans = 128, Relaxation delay = 2.5s. **HRESI-MS (m/z):** [M-H]$^-$ calcd. for $C_{19}H_{19}O_9$, 421.0776; found, 421.0786.

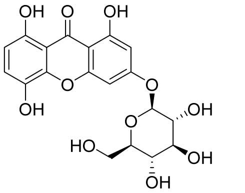


2-1b

### 3-*O*-β-D-glucosyl-1,5,8-trihydroxyxanthone(2-1b)

**$^1$H NMR (DMSO-d$_6$+D$_2$O [10:1], 500 MHz):** δ 7.27 (d, *J* = 8.9 Hz, 1H), 6.72 – 6.64 (m, 2H), 6.47 (s, 1H), 5.11 (d, *J* = 7.5 Hz, 1H), 3.70 (s, 1H), 3.55 – 3.42 (m, 2H), 3.39 – 3.26 (m, 2H), 3.19 (s, 1H). Parameters: Pulse sequence = zg30, Number of scans = 8, Relaxation delay = 1s. **HRESI-MS (m/z):** [M-H]$^-$ calcd. for $C_{19}H_{19}O_9$, 421.0776; found, 421.0760.

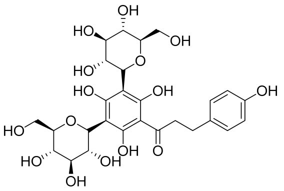


**1-1a**

1-1a:

**$^1$H NMR (DMSO-$d_6$+$D_2O$ [10:1], 600 MHz)** δ 7.06 – 7.00 (m, 1H), 6.70 – 6.65 (m, 1H), 4.71 (d, *J* = 9.8 Hz, 1H), 3.63 (d, *J* = 10.6 Hz, 1H), 3.47 (t, *J* = 9.4 Hz, 1H), 3.34 – 3.30 (m, 1H), 3.29 – 3.24 (m, 3H), 2.79 (t, *J* = 7.7 Hz, 1H). Parameters: Pulse sequence = zg30, Number of scans = 32, Relaxation delay = 1s.

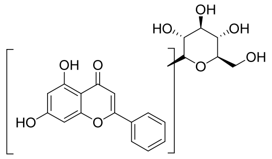


1-4a:

**1H NMR (DMSO-$d_6$+$D_2O$ [10:1], 600 MHz)** δ 7.93 – 7.90 (m, 2H), 7.51 – 7.48 (m, 2H), 7.45 – 7.39 (m, 2H), 6.67 (s, 1H), 6.41 (d, *J* = 1.7 Hz, 1H), 6.28 (d, *J* = 1.7 Hz, 1H), 5.07 (d, *J* = 7.1 Hz, 1H), 3.69 (d, *J* = 10.2 Hz, 1H), 3.52 – 3.49 (m, 1H), 3.38 – 3.36 (m, 1H), 3.33 (dd, *J* = 7.8, 6.3 Hz, 2H), 3.24 – 3.22 (m, 1H). Parameters: Pulse sequence = zg30, Number of scans = 32, Relaxation delay = 1s.

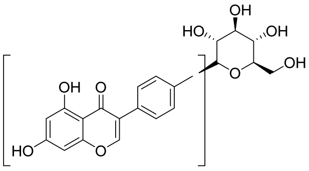


1-11a:

**$^1$H NMR (DMSO-$d_6$+$D_2O$ [10:1], 600 MHz)** δ 8.46 (d, *J* = 1.3 Hz, 1H), 7.49 – 7.46 (m, 2H), 7.27 (d, *J* = 7.9 Hz, 2H), 6.75 (d, *J* = 2.2 Hz, 1H), 6.50 (d, *J* = 2.2 Hz, 1H), 5.06 (d, *J* = 7.6 Hz, 1H), 3.73 – 3.69 (m, 1H), 3.48 – 3.44 (m, 2H), 3.34 – 3.26 (m, 2H), 3.18 (d, *J* = 9.0 Hz, 1H), 2.35 (s, 3H). Parameters: Pulse sequence = zg30, Number of scans = 24, Relaxation delay = 1s.

## 3. Copies of $^{1}$H NMR, ROESY, and $^{13}$C NMR spectra

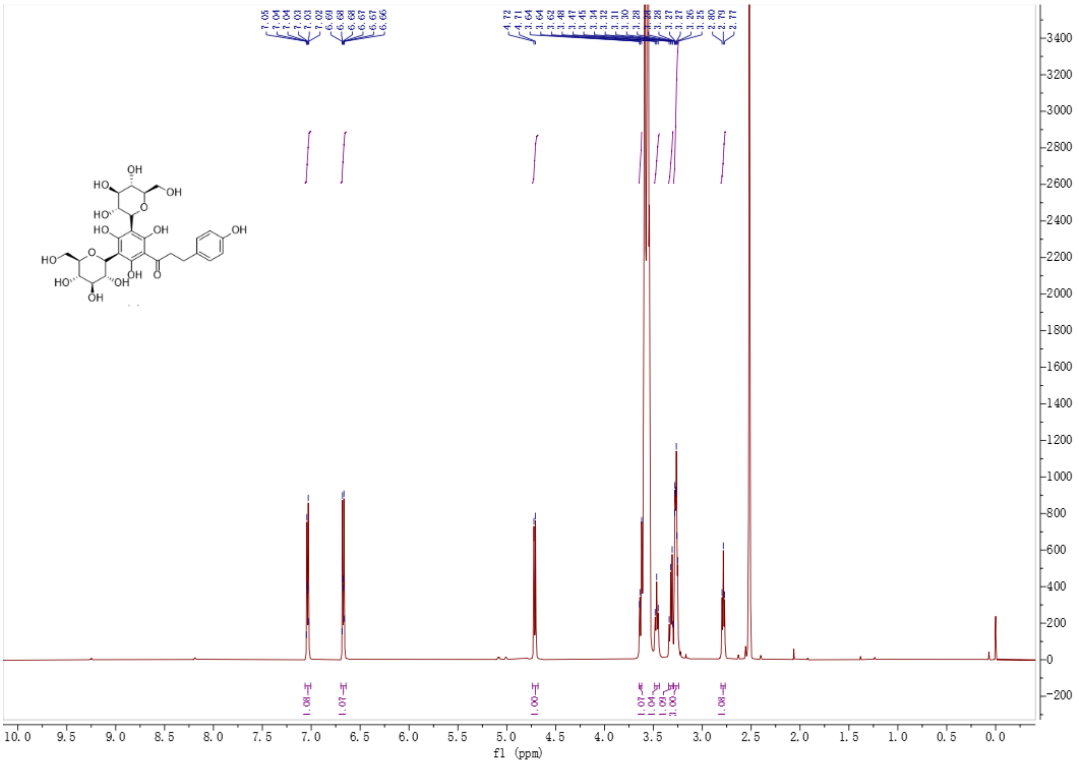

Figure S9 $^{1}$H NMR spectrum of **1-1a** (DMSO-d6+$D_2O$ [10:1], 600 MHz)

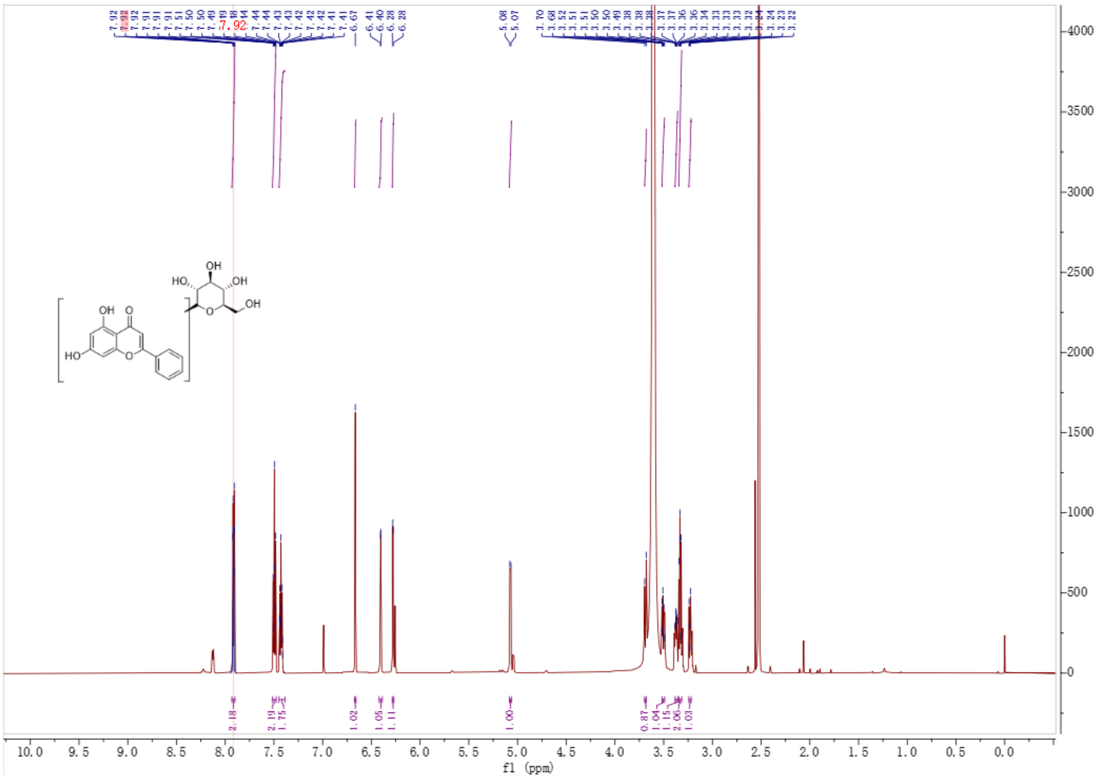

Figure S10 $^{1}$H NMR spectrum of **1-4a** (DMSO-d6+$D_2O$ [10:1], 600 MHz)

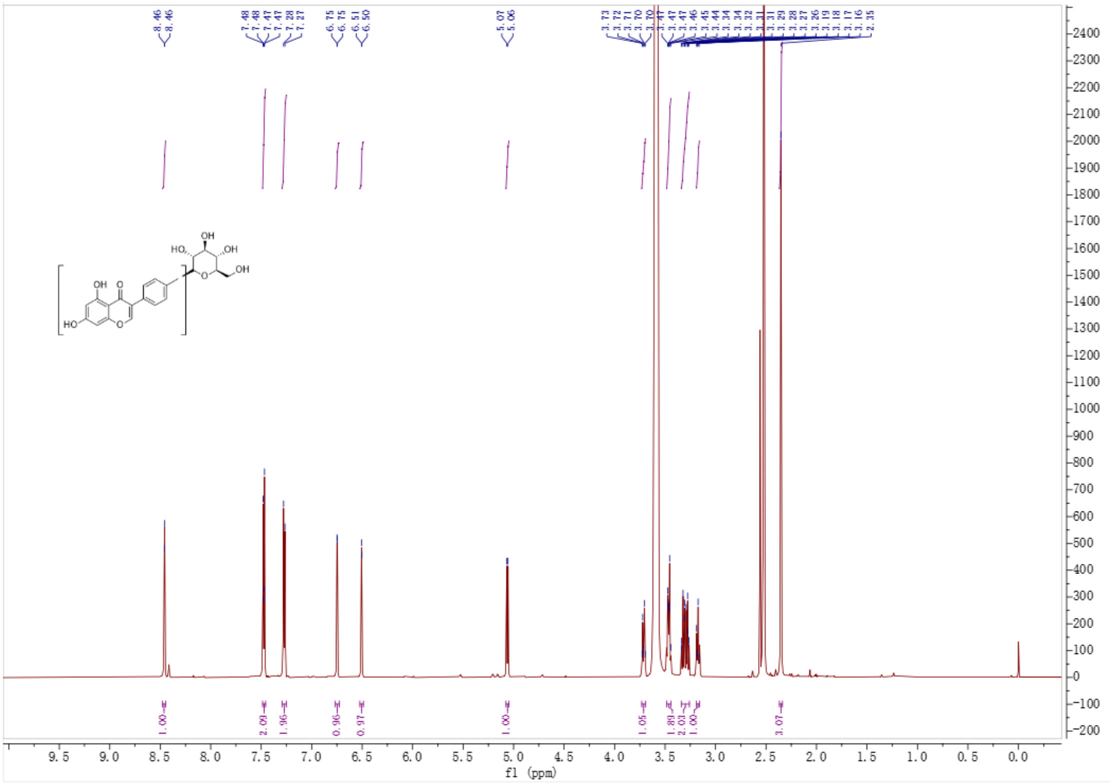

Figure S11 $^1$H NMR spectrum of **1-11a** (DMSO-d6+$D_2O$ [10:1], 600 MHz)

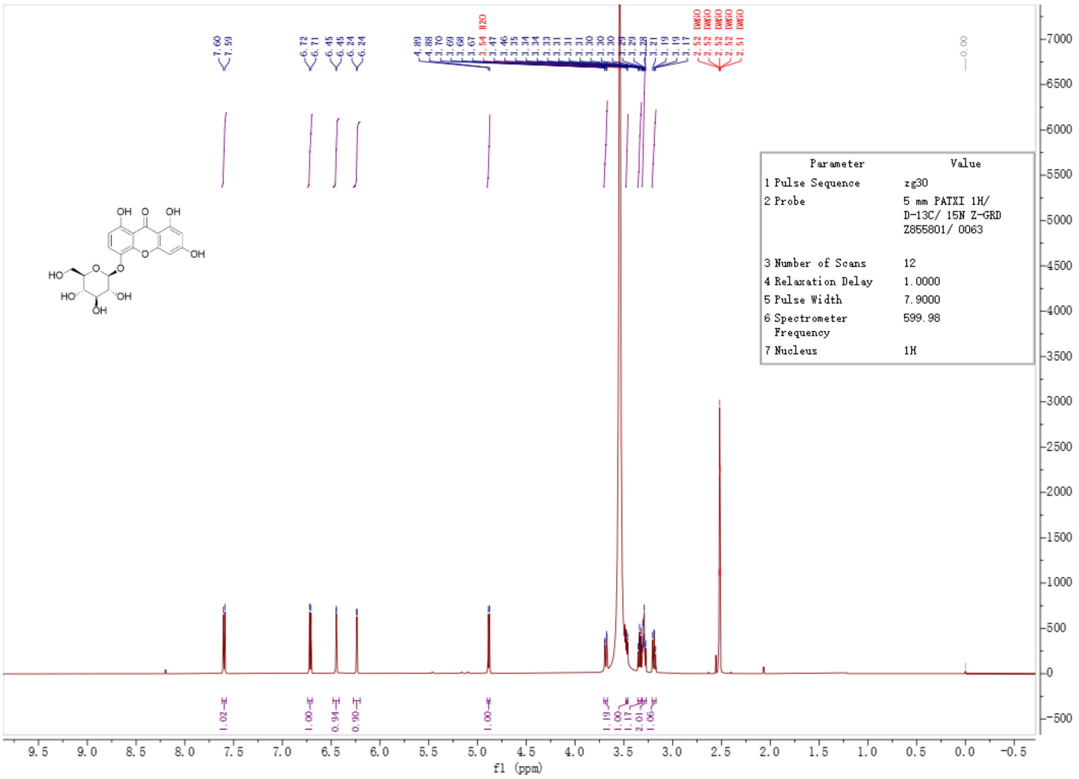


Figure S12 $^1$H NMR spectrum of **2-1a** (DMSO-d6+$D_2O$ [10:1], 600 MHz)

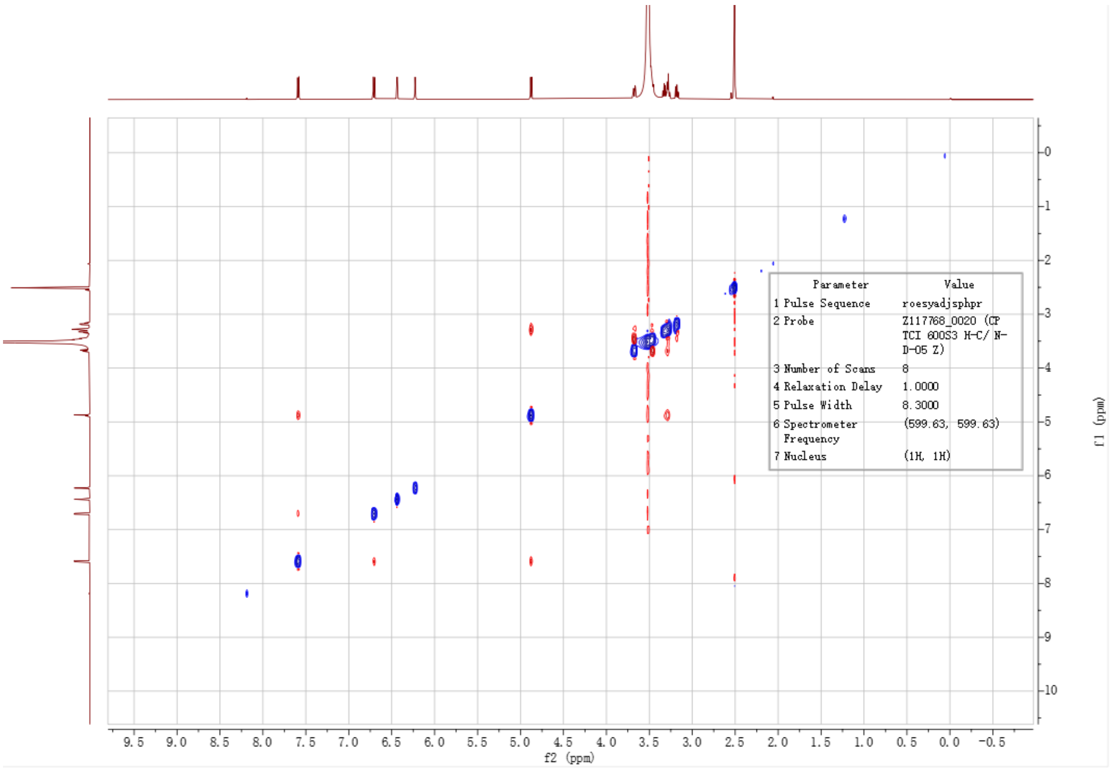


Figure S13 ROESY spectrum of **2-1a** (DMSO-d6+$D_2O$ [10:1], 600 MHz)

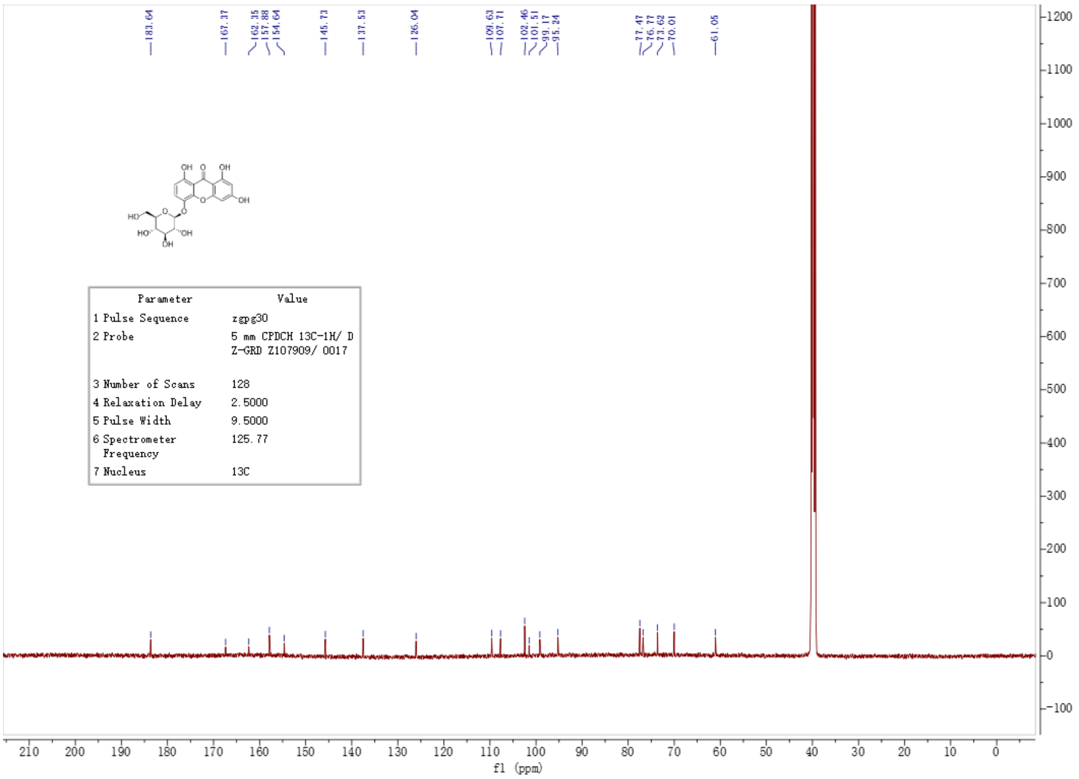


Figure S14 $^{13}$C NMR spectrum of **2-1a** (DMSO-d6, 126 MHz)

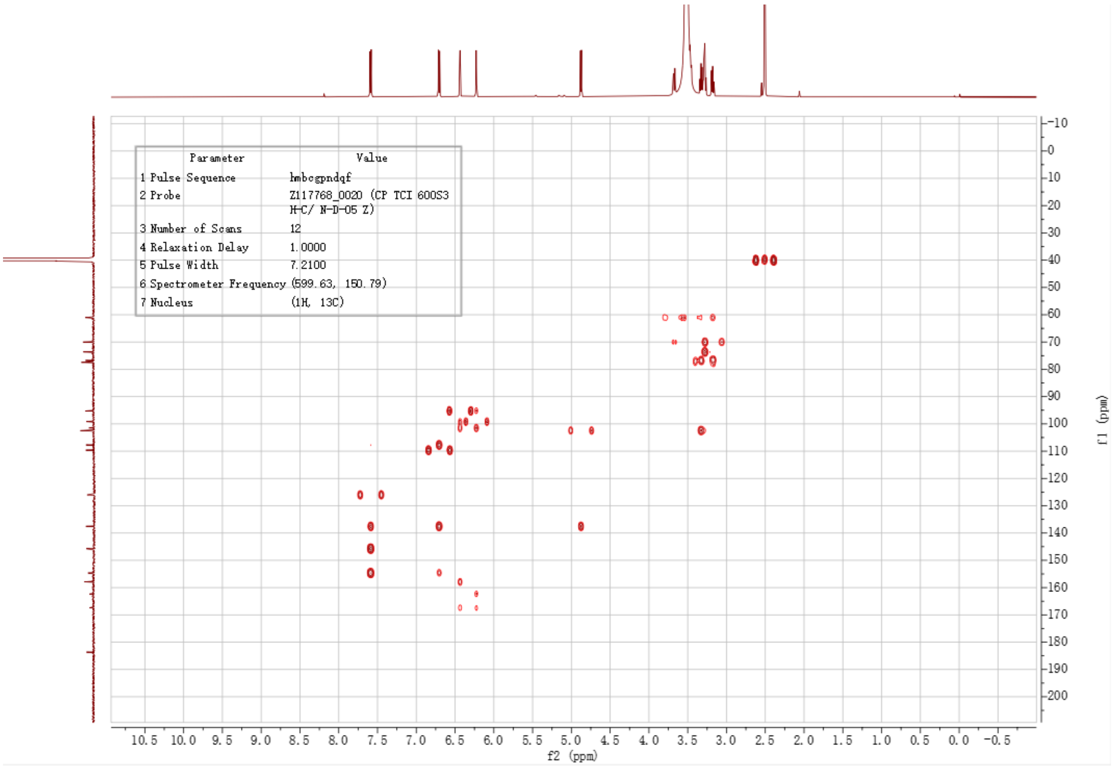


Figure S15 HMBC spectrum of **2-1a** (DMSO-d6+D2O [10:1], 600 MHz)

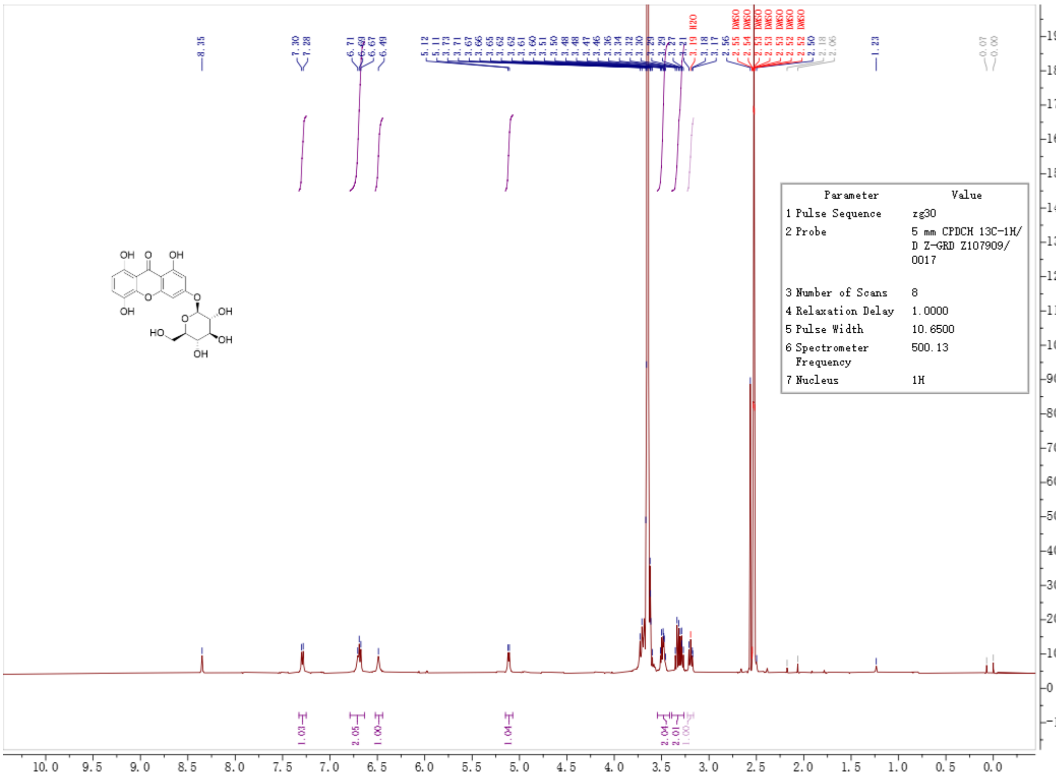


Figure S16 $^{1}$H NMR spectrum of **2-1b** (DMSO-d6+D2O [10:1], 500 MHz)

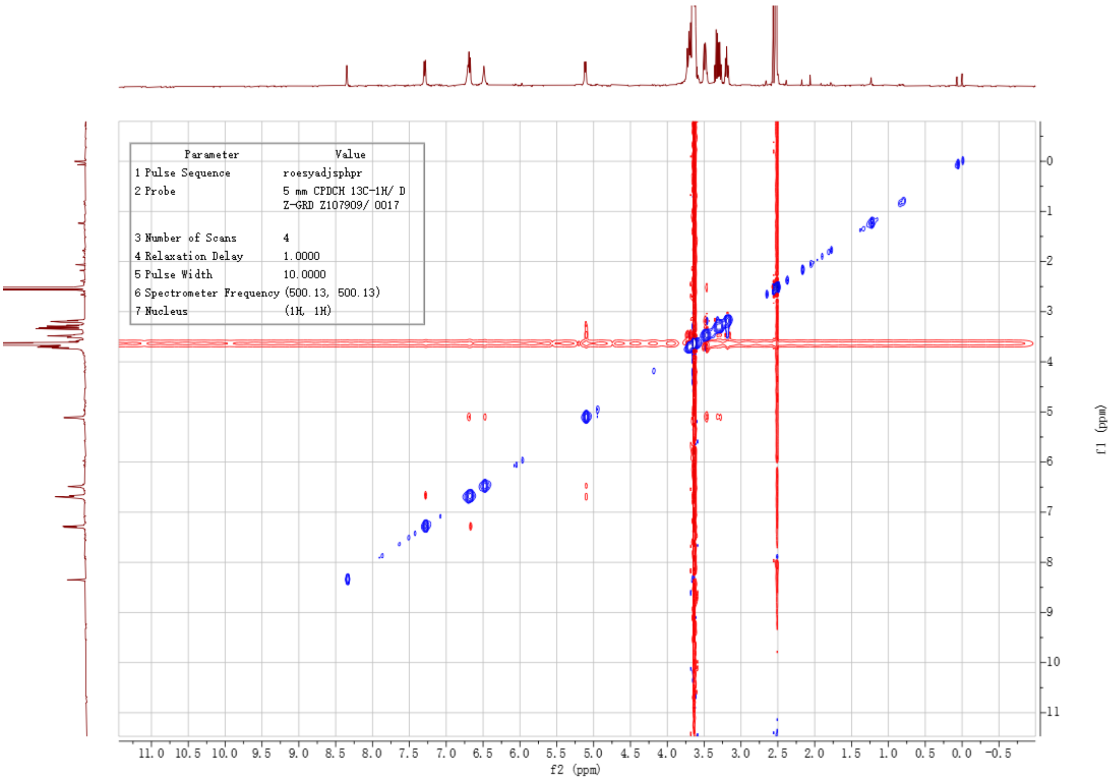


Figure S17 ROESY spectrum of **2-1b** (DMSO-d6+D2O [10:1], 500 MHz)

## 4. MS-MS spectrum of glycosylated products

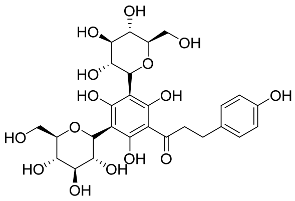

**1-1a**

Exact Mass: 598.19

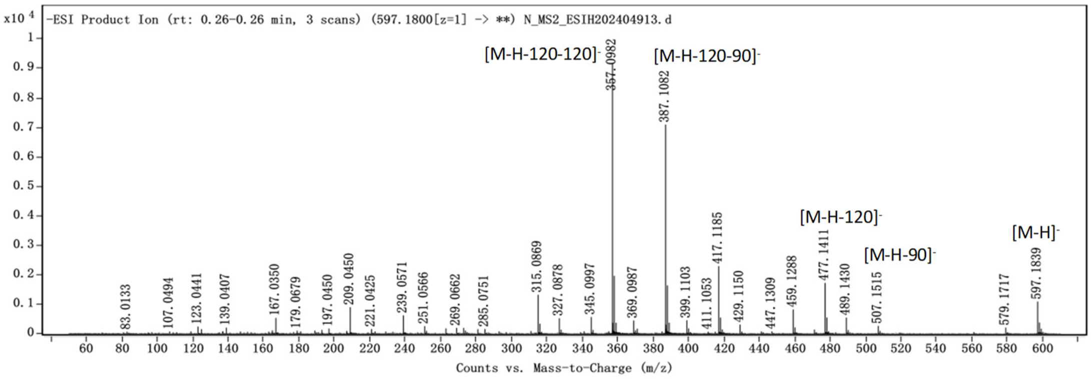


Figure S18 Typical negative $MS^2$ spectrum of **1-1a ([M-H]$^-$ 597)**.

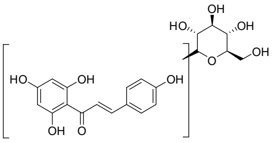

**1-3a**

Exact Mass: 434.12

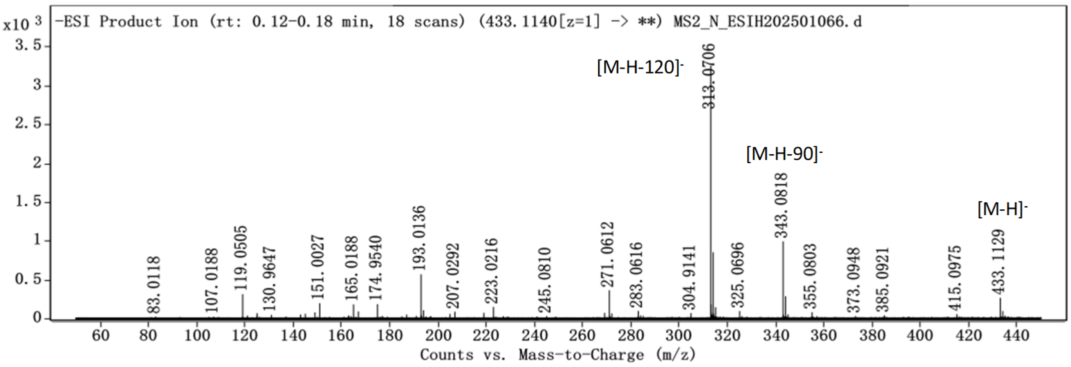


Figure S19 Typical negative MS$^2$ spectrum of **1-3a ([M-H]$^-$ 433)**.

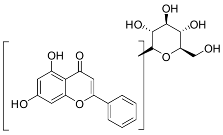

**1-4a**

Exact Mass: 416.11

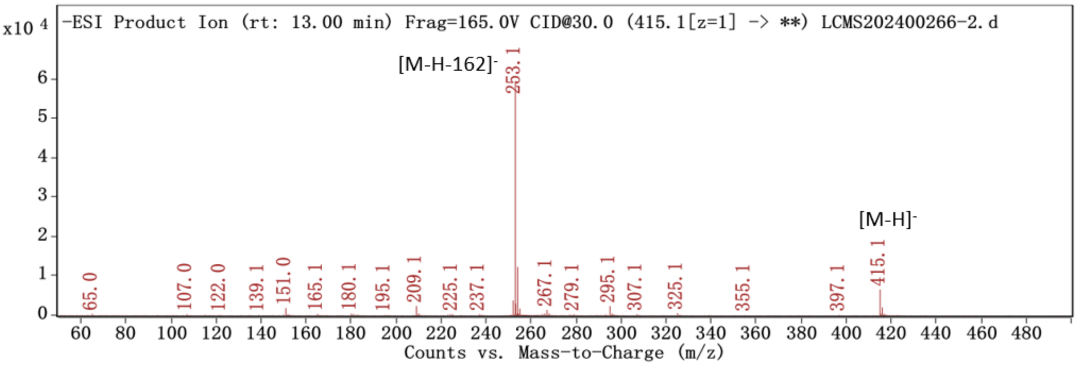


Figure S20 Typical negative MS$^2$ spectrum of **1-4a ([M-H]$^-$ 415)**.

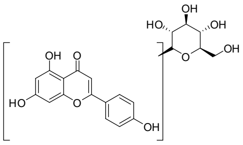

**1-5a**

Exact Mass: 432.11

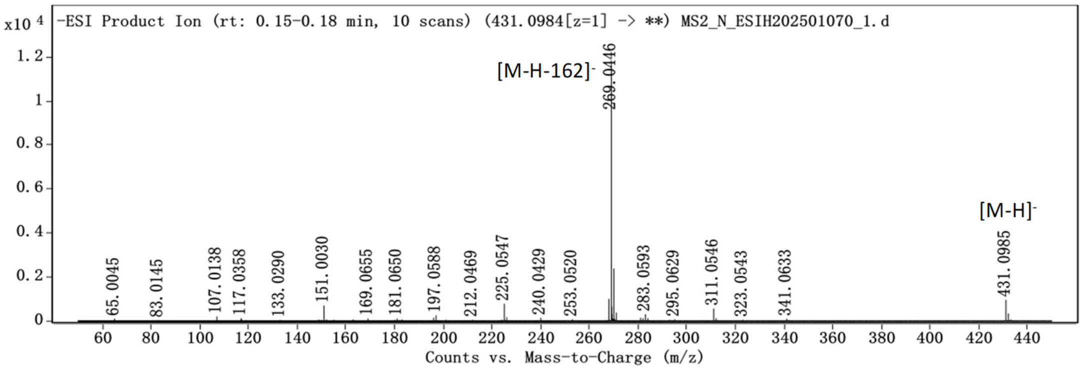


Figure S21 Typical negative MS$^2$ spectrum of **1-5a ([M-H]$^-$ 431)**.

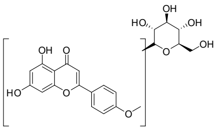


1-6a
Exact Mass: 446.12

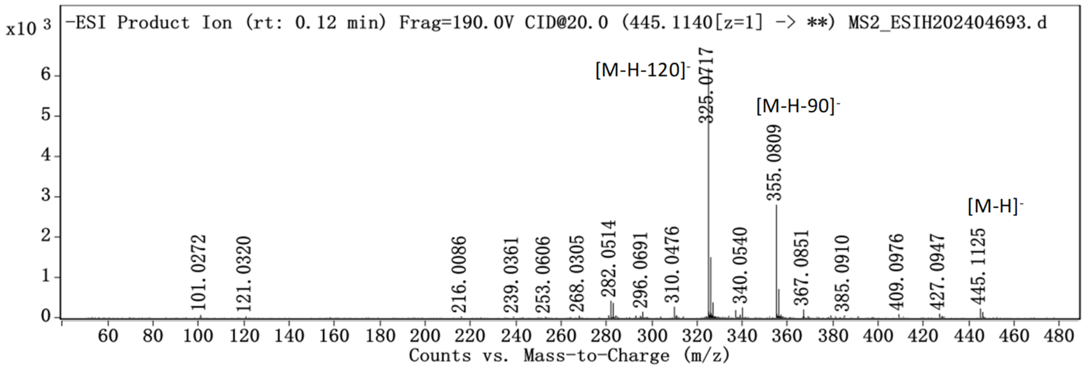


Figure S22 Typical negative MS$^2$ spectrum of **1-6a ([M-H]$^-$ 445)**.

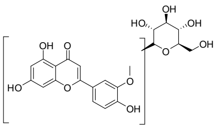


1-7a
Exact Mass: 462.12

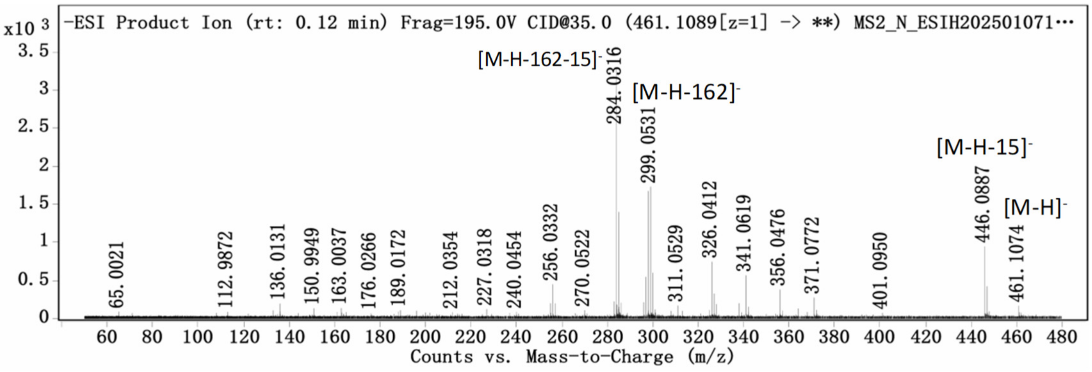


Figure S23 Typical negative MS$^2$ spectrum of **1-7a ([M-H]$^-$ 461)**.

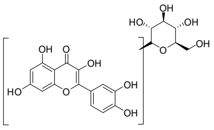

1-8a
Exact Mass: 464.10

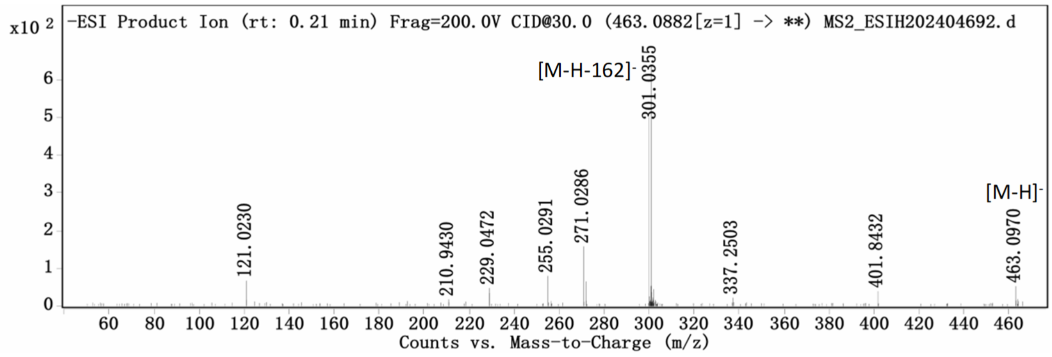


Figure S24 Typical negative MS$^2$ spectrum of **1-8a ([M-H]$^-$ 463)**.

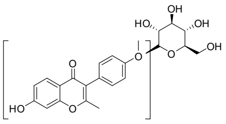

1-9a
Exact Mass: 444.14

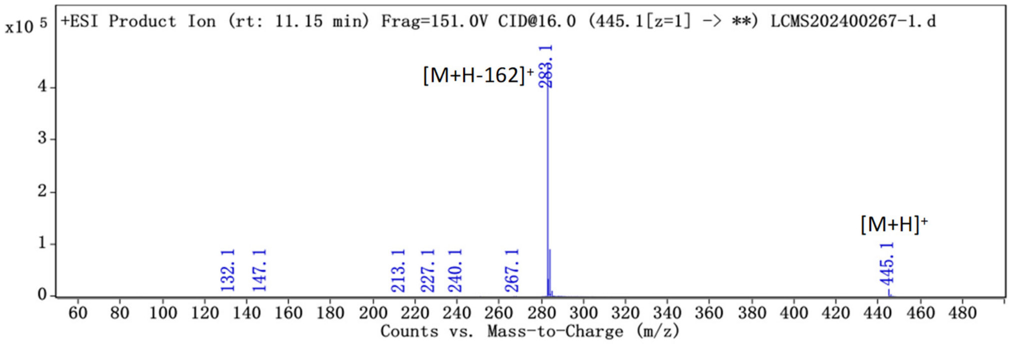


Figure S25 Typical positive MS$^2$ spectrum of **1-9a ([M+H]$^+$ 445)**.

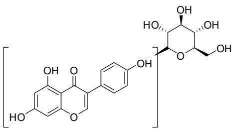

1-10a
Exact Mass: 432.11

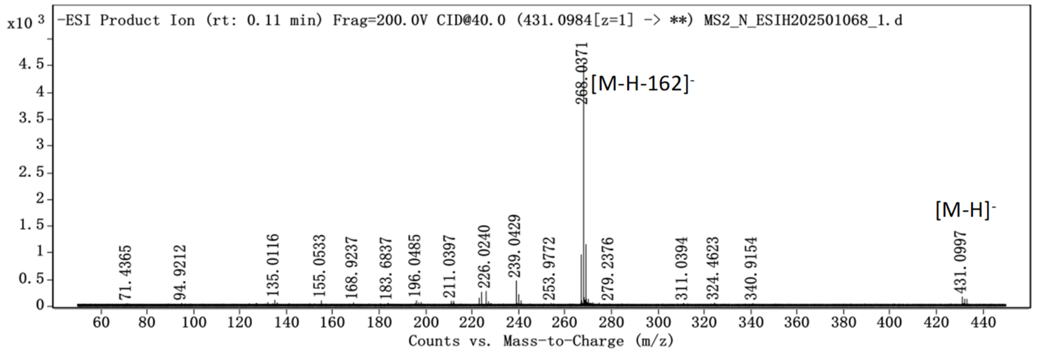


Figure S26 Typical negative MS$^2$ spectrum of **1-10a ([M-H]$^-$ 431)**.

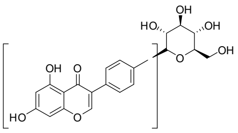


1-11a
Exact Mass: 430.13

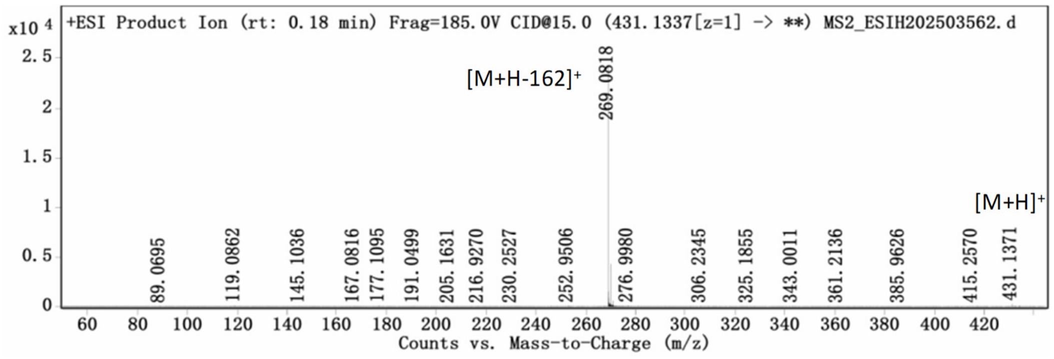


Figure S27 Typical positive MS$^2$ spectrum of **1-11a([M+H]$^+$ 431)**.

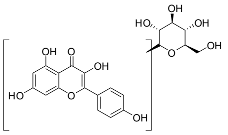


1-12a
Exact Mass: 448.10

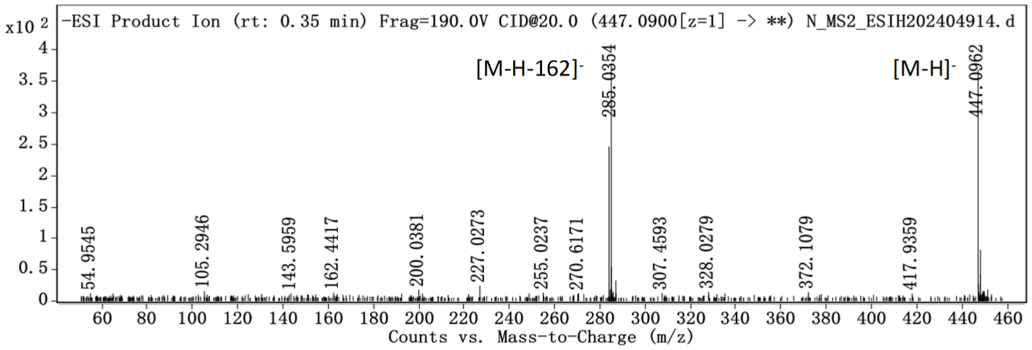


Figure S28 Typical negative MS$^2$ spectrum of **1-12a([M-H]$^-$ 447)**.

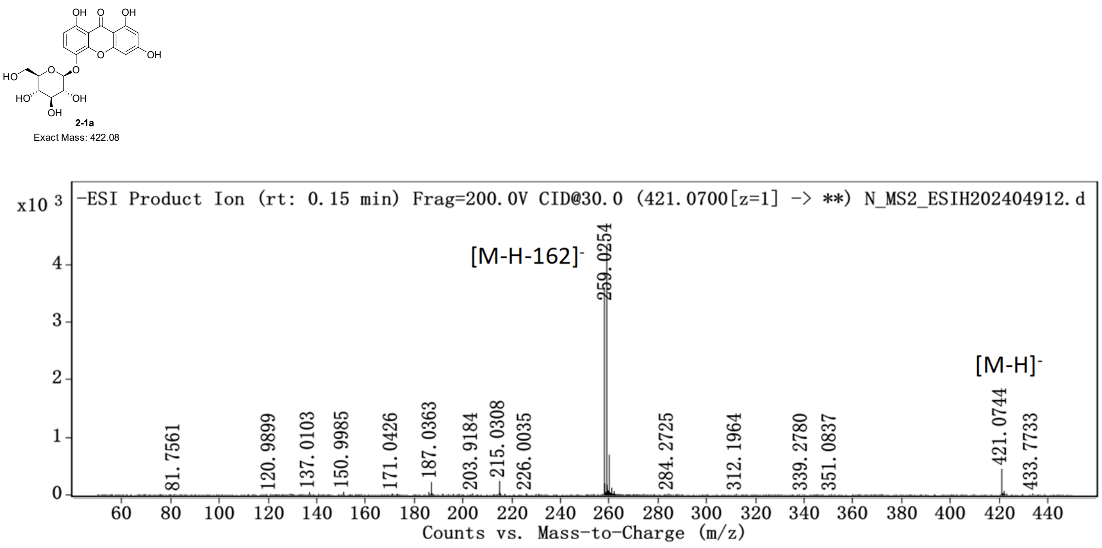


Figure S29 Typical negative MS$^2$ spectrum of **2-1a ([M-H]$^-$ 421).**

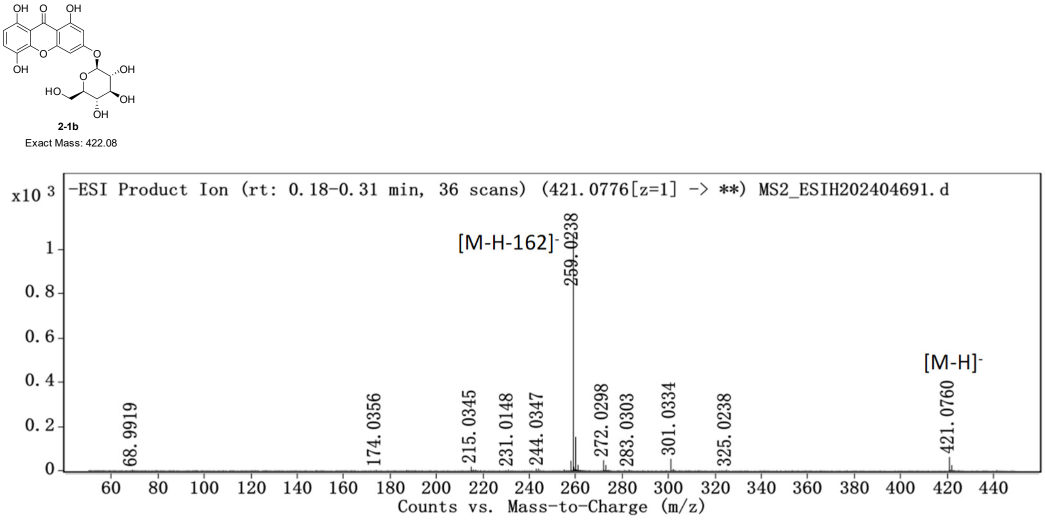


Figure S30 Typical negative MS$^2$ spectrum of **2-1b ([M-H]$^-$ 421).**
